# Enhancing cardiorespiratory fitness and quality of life in high-grade glioma through an intensive exercise intervention during chemotherapy: Proof of concept

**DOI:** 10.1093/neuonc/noaf176

**Published:** 2025-07-25

**Authors:** Johanna Jost-Engl, Ralf Ketter, Ralf Brandt, Klaus Völker, Joachim Gerß, Kathleen Jetschke, Carolin Weiss Lucas, Freerk T Baumann, Philipp M Lepper, Steffi Urbschat, Walter Stummer, Rainer Wiewrodt, Dorothee Wiewrodt, MMH Trial Investigators, Dorothee Wiewrodt, Johanna Jost-Engl, Ralf Brandt, Maren Kloss, Nora Hansel, Rainer Wiewrodt, Johanna Jost-Engl, Leonora Grimm, Irmtraud Früchte, Klaus Völker, Ross Julian, Lothar Thorwesten, Joachim Gerß, Andreas Faldum, Ralf Ketter, Steffi Urbschat, Joachim Oertel, Johanna Jost-Engl, Gabi Franke, Philipp Lepper, Kathleen Jetschke, Sylvia Rekowski, Carolin Weiss Lucas, Sophia Kochs, Freerk Baumann

**Affiliations:** Pulmonary Research Division, Department of Medicine A, University Hospital, University Münster, Münster, Germany; Department of Neurosurgery, University Hospital, Saarland University, Homburg/Saar, Germany; Department of Neurosurgery, University Hospital, University Münster, Münster, Germany; Department of Neurosurgery, University Hospital, Saarland University, Homburg/Saar, Germany; Department of Neurosurgery, University Hospital, University Münster, Münster, Germany; Institute of Sports Science, University Hospital, University Münster, Münster, Germany; Institute of Biostatistics and Clinical Research, University Münster, Münster, Germany; Department of Neurosurgery, University Hospital Knappschaftskrankenhaus, Ruhr University Bochum, Bochum, Germany; Department of General Neurosurgery, Center for Neurosurgery, University Hospital of Cologne, Cologne, Germany; Department I of Internal Medicine, Center for Integrated Oncology (CIO), University Hospital of Cologne, Cologne, Germany; Department of Internal Medicine, Pneumology and Critical Care Medicine, University Hospital Bielefeld Campus Bethel and University of Bielefeld, Bielefeld, Germany; Department of Pulmonary Medicine, University Hospital, Saarland University, Homburg/Saar, Germany; Department of Neurosurgery, University Hospital, Saarland University, Homburg/Saar, Germany; Department of Neurosurgery, University Hospital, University Münster, Münster, Germany; Pulmonary Research Division, Department of Medicine A, University Hospital, University Münster, Münster, Germany; Department of Neurosurgery, University Hospital, University Münster, Münster, Germany

**Keywords:** Active in Neuro-Oncology (ActiNO), cardiopulmonary exercise testing, glioblastoma, physical activity, supportive therapy

## Abstract

**Background:**

High-grade glioma (HGG) patients experience enormous disease burden both from tumor- and treatment-related symptoms. Exercise can improve physical fitness and quality of life (QoL); yet experience in neuro-oncology, especially with high-intensity exercise, remains limited. This study evaluated feasibility, safety, and efficacy of the intensive, structured 16-week strength and endurance program, “*Act*ive *i*n *N*euro-*O*ncology” (ActiNO) for HGG patients undergoing chemotherapy.

**Methods:**

In this prospective, oligocentric, single-arm proof-of-concept trial, 54 HGG patients participated in ActiNO, with twice-weekly supervised exercise sessions. The primary endpoint was cardiorespiratory fitness, assessed via physical working capacity (PWC_75%_)—the workload (W/kg body weight) achieved at 75% of age-adjusted maximum heart rate during a maximal cardiopulmonary exercise test. Secondary endpoints included peak oxygen uptake (VO_2_peak), peak power output (Ppeak), and QoL (EORTC QLQ-C30). Analyses focused on within-subject changes from pre- to post-intervention. Additionally, comparisons to normative data were performed. Feasibility was assessed via accrual, adherence, and attrition; safety via adverse event monitoring (CTCAE).

**Results:**

Program tolerance was high, with few exercise-related adverse events (all CTCAE grade 1-2). Over 16 weeks, significant improvements were observed in PWC_75%_ (1.023-1.256 W/kg BW, +23%), VO_2_peak (23.04-26.09 ml/min/kg BW, +13%), and Ppeak (1.771-2.104 W/kg BW, +19%). QoL, including global health and physical functioning, improved, reaching normative values. Adherence was high (85%), though attrition was 48%, mainly due to disease progression or physical constraints.

**Conclusions:**

High-intensity exercise is feasible and safe in HGG patients undergoing chemotherapy. The observed improvements in physical fitness and QoL support incorporating structured exercise into neuro-oncology care.

**Key points**

- Supervised high-intensity exercise in high-grade glioma (HGG) patients is feasible and safe.
- Exercise significantly improved physical fitness and quality of life in HGG patients under chemotherapy.

**Importance of the Study**

Despite increasing evidence supporting the benefits of exercise in oncology, research on structured, high-intensity training for high-grade glioma patients remains scarce. This study demonstrates that supervised exercise during adjuvant chemotherapy is not only safe but also significantly improves physical fitness and quality of life. We provide detailed maximal cardiopulmonary exercise testing data and assess adherence, feasibility, and safety comprehensively. Our findings suggest that structured exercise may be a promising approach to both mitigate physical decline and enhance quality of life in HGG patients; however, randomized controlled trials are needed to confirm these effects. Furthermore, such future trials should also refine exercise protocols to define optimal training parameters and explore potential survival benefits.

Despite their relatively low incidence, brain tumors—predominantly gliomas as the most common primary form—carry a high disease burden characterized by considerable mortality and a variety of symptoms, both due to the tumor and its multimodal treatments.^1–3^ Therapeutic strategies aim to prolong survival while maximizing quality of life (QoL) by mitigating common symptoms, encompassing physical, cognitive, and psychological domains, collectively deteriorating overall QoL.^4,5^

Studies in glioma patients, including our own, suggest that exercise mitigates some of these issues.^6–12^ Higher cardiorespiratory fitness and self-reported exercise behavior have been correlated with improved QoL domains in initial studies.^9,13–15^ While exercise has gained traction in general oncology,^16,17^ its integration into neuro-oncology remains limited. Concerns about safety persist among both patients and healthcare providers. A national guide on exercise in cancer care cautions against intensive exercise in brain tumor patients due to potential risks such as neurological episodes or sudden loss of consciousness.^18^ Consequently, most fitness assessments rely on submaximal testing in brain tumor patients,^9,13^ while the supporting evidence for exercise interventions in this group is rated as weak.^6^ These uncertainties may contribute to the reduced physical performance observed compared to normative data.^13–15,19–21^

Since 2011, upon repeated requests from our patients, we have implemented exercise programs for brain tumor patients, which led to the development of the “Active in Neuro-Oncology” (ActiNO) concept.^7^ In this prior retrospective study involving 45 glioma patients (58% glioblastoma (GBM), 76% undergoing adjuvant therapy), participants demonstrated a mean 11.5% improvement in physical working capacity (PWC_75%_) after a median of 16 training sessions.^7^ For safety reasons, PWC_75%_ was applied back then.

PWC_75%_ is a validated, submaximal cycle ergometry-based measure of aerobic capacity that estimates the workload (in W/kg body weight) achieved at 75% of an individual’s predicted maximum heart rate (HR).^22,23^ Unlike gold standard measures such as peak oxygen uptake (VO_2_peak) or peak power output (Ppeak), PWC does not require participants to reach total exhaustion, yet still detects changes in aerobic capacity. It is easy to implement as it does not require sophisticated cardiopulmonary exercise testing (CPET) equipment, which is usually available in tertiary medical centers only. PWC_75%_ has been widely used in exercise physiology and population-based studies,^22–27^ with age and sex-specific normative data available. Therefore, we selected PWC_75%_ as the primary endpoint complemented by VO_2_peak and Ppeak as secondary endpoints.

We hypothesized that a mean ≥15% improvement in PWC_75%_ would be feasible and clinically relevant, based on: (1) our previous data,^7^ assuming the longer intervention period and the greater number of training sessions in this highly structured, prospective design, and (2) data from randomized controlled exercise trials in various cancer types^28^ linked to established reference values.^29^ For methodological rationale, see Supplementary Content 1. This paper presents our prospective, oligocenter trial evaluating the feasibility, safety, and efficacy of a 16-week supervised, intensive strength and endurance training program aimed at improving physical performance and QoL in high-grade glioma (HGG) patients undergoing adjuvant chemotherapy.

## Material and Methods

### Design

This proof-of-concept study is an oligocenter, prospective, clinical, single-arm interventional trial conducted at the University Hospitals of Münster, Saarland, Bochum, and Cologne (July 2020-October 2024). It investigates the impact of the 16-week exercise program, “ActiNO,”^7^ specifically developed for brain tumor patients, on physical performance in HGG patients undergoing adjuvant chemotherapy. The study was approved by the respective ethics committees (Primary site: Westphalian-Lippe 2015-087-f-S; Clinical Trials ID: NCT05015543), and all patients provided informed consent.

### Participants

The study included newly diagnosed malignant glioma (WHO grade 3 or 4) patients with completed surgical and concomitant radiochemotherapeutic treatments and undergoing adjuvant chemotherapy. WHO grade served as the primary inclusion criterion, reflecting standard treatment decisions at study initiation (2020). Molecular characterization according to the 2021 WHO classification (ie, IDH mutation status, O-6-methylguanine-DNA-methyltransferase (MGMT) promoter methylation)^30^ was recorded. An important inclusion criterion was a Karnofsky Performance Status (KPS) of ≥70 or Eastern Cooperative Oncology Group Performance Status (ECOG) <2. This was chosen to ensure participants could safely complete the planned intensive exercise intervention and CPET assessments. Main exclusion criteria that would interfere with participants’ ability to safely complete assessments or exercise sessions, were diagnosed dementia (Mini-Mental State Examination score <24/30) and contraindications to intensive exercise, including maximal CPET. Following standardized assessment, CPET eligibility was finally cleared by a board-certified pulmonologist, ensuring that only patients with well-controlled comorbidities underwent CPET. A full list of eligibility criteria is provided in Supplementary Content 2.

### Exercise Intervention and Study Procedure

The intervention followed the “ActiNO” program,^7^ a supervised, individualized training concept for brain tumor patients. Participants attended 2 in-person exercise sessions per week for 16 weeks (50 min each) at the respective clinics or cooperating training centers nearby, equipped with cycle ergometers and resistance machines. Training was delivered 1:1 by certified sports scientists or physiotherapists trained in the standardized ActiNO protocol. Sessions alternated between endurance and strength training. Endurance sessions used stationary ergometers, targeting an average of 75% of the highest previously recorded HRmax, and included intervals and coordinative elements.

HR was continuously monitored (Polar M430 incl. HR sensor H7/9, Kempele, Finland). The individual workload, ie, intensity, was adjusted dynamically to maintain the target HR, accounting for day-to-day conditions and avoiding overexertion. Internal load (HR response) guided exercise intensity; external load (wattage) was secondary and adjusted accordingly, enabling gradual performance increases without increasing perceived exertion. A representative example of a complete endurance training session, including an HR diagram, is shown in Supplementary Figure 1.

**Figure 1.**
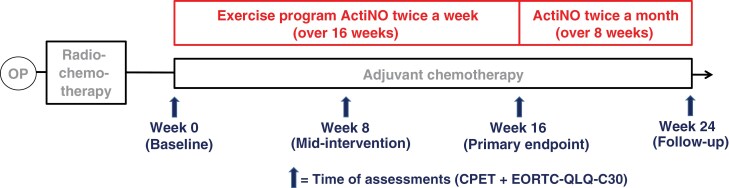
Study Timeline and Key Assessments. At study inclusion, prior to beginning the ActiNO program, participants underwent a maximal CPET to determine their current cardiorespiratory fitness. This test was repeated at 8 weeks (mid-intervention), 16 weeks (primary endpoint), and 24 weeks (follow-up) to detect changes in patients’ physical performance over time. At each of these testing points, the EORTC QLQ-C30 questionnaire was completed prior to the CPET to evaluate the participants’ QoL. Between weeks 16 and 24, a supervision phase included bimonthly personal exercise sessions to address any questions arising from participants’ continuation with independent training.

Progressive strength training targeted large muscle groups using various resistance machines and was conducted at a moderate-to-high perceived exertion level (rate of perceived exertion, 14-17/20). For more details, refer to Jost et al.^7^ The study procedure is illustrated in Figure 1.

After 16 weeks, patients entered an 8-week follow-up phase (week 16-24), during which they were encouraged to remain active independently. To support this, 2 supervised sessions per month (1 endurance, 1 strength, following the ActiNO concept) were offered. These were not intended to further increase fitness, but rather to support the transition to independent training and provide feedback. A final assessment (week 24) was conducted to evaluate whether patients’ fitness and QoL levels were sustained.

### Criteria for Premature Study Termination

All eligibility criteria remained valid throughout study participation and guided any premature termination. Continuation was carefully reviewed by a multidisciplinary team (neurosurgeons, sports scientists, psycho-oncologists, and internists), with safety and patient well-being always prioritized. Every effort was made to enable patients to complete the study protocol, or to continue as long as possible. Regarding adverse events (AE), patients were excluded only if the event impaired their ability to adhere to the protocol.

In cases of radiologically confirmed progression, patients without clinical deterioration who wished to continue were allowed to remain in the study. If progression was accompanied by clinical decline, patients were excluded. Upon patient request, these patients were offered alternative training within institutional supportive care programs, provided safety could be ensured. They were no longer part of the study and not followed for further outcome measures.

### Feasibility and Safety

Feasibility was assessed using 3 key metrics and was determined for each participant throughout their study participation: accrual, adherence, and attrition. Accrual reflected the proportion of eligible patients who enrolled, as already discussed in our previous publication,^20^ with a target of ≥25% of all eligible patients. Adherence was evaluated by (1) the percentage of completed sessions relative to the total number of scheduled sessions, with a target of ≥80%, and (2) the average HR during endurance sessions as a surrogate marker for training intensity, aiming for an average of 75% of HRmax; ≥70% was considered sufficient. HRmax was calculated using the equation by Tanaka et al.^31^ This standardized approach was chosen over individually measured HRmax values to minimize day-to-day variability and ensure a consistent training intensity across participants. In patients on HR-regulating agents (eg, beta-blockers), the age-based HRmax usually cannot be reached. For these patients, HRmax was defined by the peak HR during initial CPET. Lastly, attrition was defined as the drop-out rate (aim < 30%).

Safety assessment involved systematically categorizing AEs occurring around training sessions and CPETs according to the Common Terminology Criteria for Adverse Events (CTCAE), version 5.0. Each AE was also evaluated for its relation to the intervention (unrelated, possibly related, or definitely related). Symptoms clearly attributable to chemotherapy (eg, thrombocytopenia) or planned hospitalizations (eg, for diagnostic purposes; unrelated to the exercise intervention) were not classified as AEs. Preexisting symptoms, documented at baseline, were only recorded as AEs if they deteriorated during the study period. Additionally, progression rates both during active study participation and within 12 months post-diagnosis were determined and compared to external datasets^32,33^ to evaluate any potential risk of accelerated disease progression due to the intervention.

### Cardiorespiratory Fitness Assessment

Cardiorespiratory fitness changes were assessed via maximal CPETs, following the procedure thoroughly described in our previous publication.^20^ CPETs were always conducted at the same time of the day by the same personnel. Key parameters for the analysis included VO_2_peak, Ppeak, PWC_75%_, and power output at a respiratory exchange ratio (RER) of 1.0. VO_2_peak and Ppeak results were further compared to age-, weight-, height-, and sex-adjusted normative values (SHIP study,^34^), while PWC_75%_ results were compared to normative values based on the DEGS1 study.^23^ To explore predictors of intervention response, multivariate analyses were conducted using various baseline characteristics, including data stratification by ECOG status and by physical activity behavior. The latter was derived from self-reported questionnaires^20^ and categorized patients into “stay-active” (engaged in structured exercise both before and after diagnosis), “get-inactive” (engaged in structured exercise before diagnosis but not afterward), and “all-inactive” (did not engage in structured exercise either before or after diagnosis).

### Quality of Life Assessment

As secondary endpoints, several standardized patient-reported outcomes and cognitive tests (NOA-19 study’s test battery^35^) were collected. This paper focuses exclusively on QoL data derived from the European Organisation for Research and Treatment of Cancer—Quality of Life Questionnaire Core 30 (EORTC QLQ-C30), a 30-item instrument assessing cancer-related QoL, including global health and physical functioning, which are the focus of the QoL analysis presented here. Scores were converted to a 0-100 scale (higher = better QoL) and compared to age-matched normative values.^36^ Questionnaires were self-administered with study nurse support if needed. Due to the breadth of collected data and in order to maintain focus, cognitive data and additional QoL domains will be presented in a separate, forthcoming publication.

### Statistics

Sample size calculation: Based on our previous work,^7^ we anticipated a 15% improvement in the primary endpoint (PWC_75%_) at week 16 (mean from 1.5 ± 0.5 to 1.725 ± 0.5 W/kg, intraindividual correlation 0.5). Using G*Power 3.1. (two-sided paired *t*-test, α=0.05, power = 80%), a sample size of *n* = 50 participants actually starting the training program was deemed sufficient.

The primary analysis assessed intraindividual changes in outcomes (PWC_75%_, VO_2_peak, Ppeak, global health, and physical functioning) between baseline and week 16. Additional comparisons were conducted for mid-intervention (week 8) and follow-up (week 24) values. Furthermore, comparisons to normative data of these parameters were assessed at baseline and at week 16.

Statistical analyses: Standard descriptive analyses and the Shapiro-Wilk test for normality were applied. For continuous variables, means and standard deviations (SD) (if normally distributed) or medians (Mdn) and interquartile ranges (IQR) (if not normally distributed) were reported. Paired t-tests or Wilcoxon tests were used accordingly, to examine changes in physical outcomes from maximal CPETs or in QoL at different time points, as well as when comparing patient scores to normative values. Correlations between QoL and fitness parameters were assessed via Spearman’s rank correlation. To explore predictors of training response, multivariate linear regression was conducted using baseline variables (eg, age, sex, ECOG status, MGMT status, and dexamethasone use). In addition, binary logistic regression models were used to explore predictors of categorical training response outcomes. All analyses were conducted with SPSS 29.0 (IBM, Armonk, NY, USA).

## Results

### Participants

A total of 54 patients (57% male, mean age 59 years, range 27-84) from 4 sites participated. Most patients suffered from GBM (93%), and over half presented with neurological impairments at baseline. Full baseline characteristics are listed in Table 1. Patients started the intervention a median of 6 weeks after completion of radiochemotherapy. At mid-intervention (week 8), all patients were still undergoing adjuvant chemotherapy; at week 16, all but 1; at the follow-up (week 24), 16 of 23 (ie, 7 had completed adjuvant treatment). Further analyses ruled out potential confounding effects of treatment completion (at week 24) or timing of inclusion on observed fitness and QoL outcomes (data not shown).

**Table 1.** Baseline Patient Characteristics

	Mean ± SD/*n* (%)
**Patients**	54 (100)
**Socio-demographic**	
Sex, male	31 (57.4)
Age, years	58.7 ± 11.2
≥65 yrs	16 (29.6)
**Clinical information**	
ECOG	
0	34 (63.0)
1	20 (37.0)
Body mass index (BMI)	25.7 ± 4.6
Tumor histology	
Glioblastoma (GBM) (IDH-wildtype)	50 (92.6)
Astrocytoma (WHO grade 4) (IDH1-mutant)	2 (3.7)
Astrocytoma (WHO grade 3) (IDH1-mutant)	2 (3.7)
MGMT promoter methylation (incl. weak methylation)	33 (61.1)
Tumor localization—laterality	
Right	26 (48.1)
Left	24 (44.4)
Both	4 (7.4)
Tumor localization—region	
Thalamus	2 (3.7)
Frontal	13 (24.1)
Temporal	7 (13.0)
Parietal	11 (20.4)
Occipital	2 (3.7)
Infratentorial	1 (1.9)
Brainstem	1 (1.9)
Multilobular	17 (31.5)
Neurological impairment due to brain tumor (multiple answers possible)	33 (61.1)
Speech	16 (29.6)
Motor	15 (27.8)
Sensory	9 (16.7)
Visual	9 (16.7)
Other brain tumor-specific symptoms (multiple answers possible)	42 (77.8)
Fatigue	33 (61.1)
Headaches	15 (27.8)
Nausea	12 (22.2)
Cognitive impairment	9 (16.7)
Dizziness	8 (14.8)
Current antiepileptic drug treatment	29 (53.7)
**Treatment information**	
Cranial surgery	54 (100)
Biopsy only	16 (29.6)
Partial resection (5%-95% tumor resection)	12 (22.2)
Total resection (≥95% tumor resection)	26 (48.1)
Time post-diagnosis, months (IQR)	3.5 (IQR: 1.5)
Time post radiotherapy, months (IQR)	1.2 (IQR: 1.1)
Current adjuvant treatment (after concurrent radiochemotherapy)	54 (100)
Temozolomide (TMZ)^22^	34 (63.0)
Lomustine-temozolomide combination^23^	17 (31.5)
Other regimens (continuous low-dose TMZ ± hydroxyurea)^a^	3 (5.6)
Additional treatment with tumor treating fields (TTF)^b^	11 (20.4)
Dexamethasone treatment at study commencement	24 (44.4)
Median dose among patients on dexamethasone [mg/day]	2 (IQR: 2.0)
**Comorbidities (permanent drug treatment; multiple answers possible)**	31 (57.4)
Cardiovascular disease^c^	25 (46.3)
Diabetes	5 (9.2)
Others^d^	21 (38.9)

^a^Continuous low-dose TMZ treatment, with or without hydroxyurea, was not administered at our institutions but was conducted by an outpatient oncologist. The regimen consisted of a daily low-dose TMZ administration of 100mg/day on a continuous basis (no interruptions planned).

^b^The specification of additional treatment with TTF is not limited to baseline characteristics but rather indicates whether patients received TTF at any point during their study participation.

^c^Cardiovascular treatments include both mono- and polytherapies (all cardiovascular drug classes, and combination therapies with diuretics, coagulation modulators, and lipid modulators).

^d^Others include hypo-/hyperthyreosis 8×, psychic disorders 4×, hormonal substitution 2× (breast cancer in remission and postmenopausal substitution), prostate hyperplasia, restless legs syndrome, hyperuricemia, bronchial asthma, and peripheral artery disease.

Of note: 6 patients had malignant diseases prior to GBM; 5 of them were cured (kidney, diagnosed 2005; prostate, diagnosed 2010 and 2019, resp.; colon, diagnosed 2003; breast, diagnosed 2005) and 1 patient was in remission (breast, diagnosed 2020). Further, 1 patient had meningioma (diagnosed >30 years prior to GBM).

### Feasibility and Safety

The recruitment process, including reasons for ineligibility and non-recruitment, has been detailed previously by Jost et al.^20^

#### Accrual.

—The recruitment rate among eligible patients was 28%. Nearly half of all ineligibility cases were due to KPS <70. Among eligible patients, logistical barriers were the leading reason for nonparticipation, accounting for almost two-thirds of cases.^20^ These findings remained unchanged in this oligocentric study (Supplementary Figure 2 for further details). Without logistical barriers (ie, travel distance to study site), the accrual rate could have exceeded 50%.

#### Adherence

.—The median training session completion rate (of patients who actually commenced training after initial CPET) was 85% (IQR: 15.5%), with 77% of patients meeting the >80% adherence threshold. Across all recorded endurance sessions, patients achieved an average HR of 75% (95% CI: 73%-77%) of HRmax, with 84% of patients reaching or exceeding the minimum target rate >70%.

#### Attrition.

**Figure 2.**
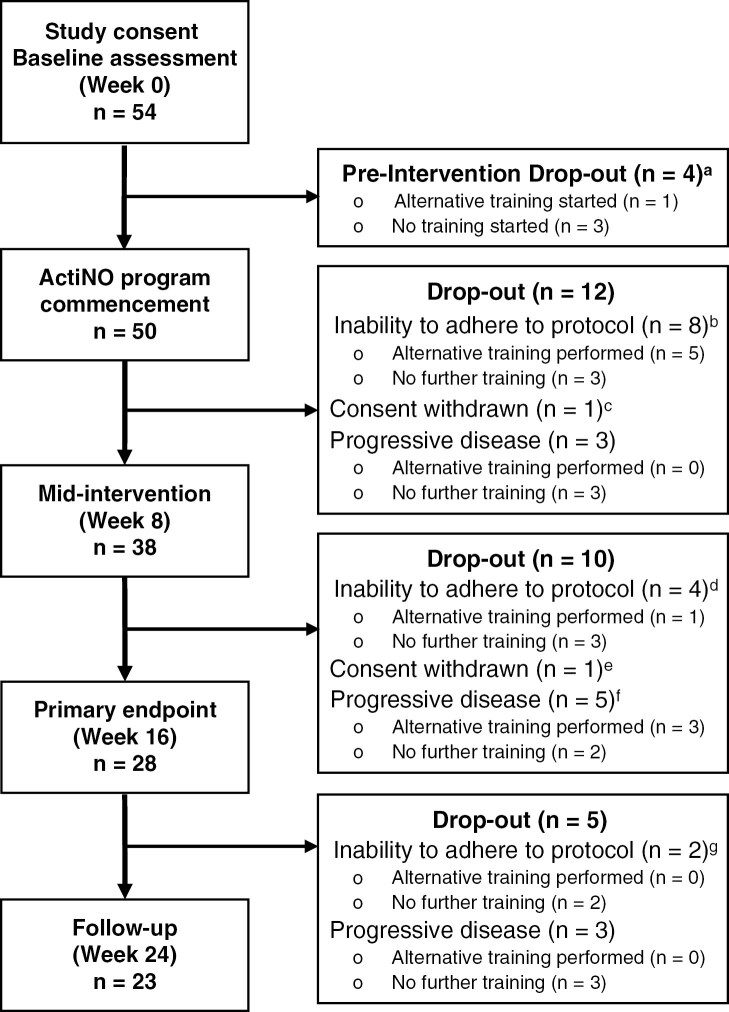
Participant Flow. The study inclusion date equals baseline CPET assessment. The “inability to adhere to protocol” category refers exclusively to patients without progressive disease. In these cases, an alternative training regimen was provided upon patient request; however, this was provided entirely outside the formal study protocol as part of the institutions’ supportive care program. It followed a less intense protocol further tailored to their specific needs but did not include any further follow-up assessments or contribute to study outcomes. Participants with progressive disease were excluded if progression was accompanied by clinical deterioration. In such cases, alternative training was only offered if explicitly requested by patients and safety was ensured. ^a^Various reasons apply: (1) Fall (prior and unrelated to first ActiNO training) leading to subsequent pain (*n* = 1); (2) Dizziness and a minor loss of balance (no fall) after initial CPET, resulting in the patient’s exclusion for safety reasons (*n* = 1); (3) Distance to the training site being too far (*n* = 1); (4) Deemed physically too unfit to endure the intensive training regimen, as evidenced by the initial CPET—training outside of study was offered and performed (*n* = 1). ^b^Various reasons apply: (1) Cardiac abnormalities leading to less intensive training for safety reasons (*n* = 2); (2) Physically too unfit to endure the intensive training regimen (*n* = 1); (3) Lack of sufficient trunk muscle strength to sit upright on the ergometer independently (*n* = 1); (4) Unable to sufficiently follow training instructions due to cognitive impairments (*n* = 1); (5) Severe infection (*n* = 1); (6) Pronounced fatigue and too distressed (*n* = 1); (7) persistent sores (buttocks) hindering training (*n* = 1). ^c^No enjoyment from training. ^d^Various reasons apply: (1) Arm fracture from a bicycle accident, unrelated to training (*n* = 1); (2) Cheekbone fracture from a fall, unrelated to training (*n* = 1); (3) COVID-19 infection (*n* = 1); (4) Clinical deterioration without progressive disease characterized by increasing motor impairments (*n* = 1). ^e^Long travel distance to training sessions was underestimated and later considered too demanding (*n* = 1). ^f^Three patients requested for and performed an alternative training program safely until their symptom burden became too severe. ^g^Various reasons apply: (1) Stroke, unrelated to training (*n* = 1); (2) Clinical deterioration without progressive disease characterized by increasing right-sided muscle weakness, especially during exertion (*n* = 1).

—By week 16, the drop-out rate was 48% (26/54, Figure 2), with the highest drop-out occurring between weeks 0 and 8 (16/54, 30%), primarily due to patients’ inability to adhere to the protocol (9/16, 56%). Patients on dexamethasone at training commencement were more likely to drop out (Supplementary Table 6, Chi²=8.98, *p* = .003), especially due to the inability to adhere (Chi²=10.656, *p* = .031, Supplementary Figures 8 and 9). Beyond week 8, disease progression became the main reason for discontinuation: 5/10 patients (50%) up to primary endpoint; 3/5 patients (60%) during follow-up (Figure 2). No drop-outs were caused by exercise-related adverse events (ERAE). While travel burden was a key reason for nonparticipation, travel time (short vs. long, median split) did not impact attrition among enrolled participants (data not shown).

#### Safety.

—In total, 64 AEs were documented in 54 patients (Table 2), with 39 patients (72%) experiencing at least one, averaging 1.2 AEs per patient during study participation (up to 24 weeks). All AEs were of CTCAE grade 1-3; no serious AE (grade 4-5) occurred, neither during training nor outside. The most frequently affected system organ class was the nervous system, followed by general disorders.

**Table 2.** Adverse Events by System Organ Class, Exercise-Relatedness, and CTCAE Severity Grades

System organ class	Any Adverse event *n* (%)^a^	Adverse event (AE)	Total AE *n* (%)^a^	Unrelated AEs			Exercise-related AEs (^◊^Possibly/^□^Definitely)		
				Grade 1 *n* (%)^a^	Grade 2 *n* (%)^a^	Grade 3 *n* (%)^a^	Grade 1 *n* (%)^a^	Grade 2 *n* (%)^a^	Grade 3 *n* (%)^a^
Nervous System	24 (44.4)	Seizure	6 (11.1)	2 (3.7)	-	4 (7.4)	-	-	-
		Dizziness^b^	5 (9.3)	-	-	-	5 (9.3)^◊^^□^	-	-
		One-sided muscle weakness	4 (7.4)	1 (1.9)	3 (5.6)	-	-	-	-
		Concentration impairment	3 (5.6)	1 (1.9)	2 (3.7)	-	-	-	-
		Cognitive disturbance	2 (3.7)	-	1 (1.9)	1 (1.9)	-	-	-
		Dysphasia	2 (3.7)	-	2 (3.7)	-	-	-	-
		Headache	1 (1.9)	1 (1.9)	-	-	-	-	-
		Stroke	1 (1.9)	-	-	1 (1.9)	-	-	-
General disorders	17 (31.5)	Disease progression^c^	11 (20.4)	n.a.			n.a.		
		Fatigue ^d^	5 (9.3)	2 (3.7)	3 (5.6)	-	-	-	-
		Localized edema (knee)	1 (1.9)	-	-	-	1 (1.9)^□^		
Infection	6 (11.1)	Upper respiratory	4 (7.4)	4 (7.4)	-	-	-	-	-
		COVID-19	1 (1.9)	1 (1.9)	-	-	-	-	-
		Other (unknown)	1 (1.9)	-	1 (1.9)	-	-	-	-
Injury	6 (11.1)	Fall	4 (7.4)	1 (1.9)	1 (1.9)	2 (3.7)	-	-	-
		Fracture	2 (3.7)	-	1 (1.9)	1 (1.9)	-	-	-
Musculoskeletal	5 (9.3)	Buttock pain^e^	3 (5.6)	-	-	-	3 (5.6)^□^	-	-
		Muscle-weakness lower limb	1 (1.9)	-	1 (1.9)	-	-	-	-
		Muscle-weakness trunk	1 (1.9)	-	1 (1.9)	-	-	-	-
Cardiac	4 (7.4)	Sinus bradycardia	2 (3.7)	-	-	-	2 (3.7)^□^	-	-
		Chest pain	1 (1.9)	-	-	-	1 (1.9)^□^	-	-
		Other: ST-segment depression^f^	1 (1.9)	1 (1.9)	-	-	-	-	-
Gastrointestinal	1 (1.9)	Vomiting	1 (1.9)	-	-	-	1 (1.9)^◊^	-	-
Respiratory	1 (1.9)	Bronchial obstruction	1 (1.9)	-	-	-	-	1 (1.9)^□^	-

Throughout the study, no patient experienced a serious adverse event grade 4 or 5, neither during training nor outside of it.

^a^Percentages are based on the total number of patients (*n* = 54, 100%), including those without any AEs, to estimate the risk of the respective AE. Some patients experienced more than 1 AE.

^b^In 2/5 patients, strength training exercises - especially when performed in a standing position (here: cable machine) - occasionally led to intermittent dizziness, which was effectively managed by incorporating seated rest periods between sets, allowing further execution to remain feasible and well-tolerated. 1/5 experienced overexertion during endurance training, 1/5 experienced dizziness following maximal CPET, and 1/5 dizziness case was associated with documented cardiac issues. However, all events resolved without medical intervention needed.

^c^Disease progression within the study protocol. In 10/11 cases (91%), progression was accompanied by clinical deterioration of general condition. In these cases, general clinical deterioration or the exacerbation of previously existing symptoms related to disease progression were not further listed in this table.

^d^Fatigue was recorded only if newly onset or deterioration of a preexisting condition.

^e^Buttock pain was observed exclusively at 1 study site and linked to the respective ergometer saddle. The ergometer was switched to prevent further discomfort.

^f^Asymptomatic and non-reproducible.

The majority of AEs (*n* = 50, 79.7%) were unrelated to exercise. 14 ERAE (possibly or definitely) were recorded (20.3%), with short, transient dizziness being most common (eg, standing up from machine after resistance exercise). The rate of ERAEs was low (20%), predominantly of grade 1 (93%; all resolving without medical intervention), except for one bronchial obstruction (grade 2, single albuterol inhalation needed). Further, all these events are typical for exercises involving maximal exertion and not uncommon to occur in a similarly distributed population without brain tumors. For further details, see Table 2 (percentages based on total number of participants) and Supplementary Table 1 (percentages based on total number of AEs).

Disease progression accounted for the highest number of AEs, reflecting its clinical significance in GBM. During the active course of study participation up to week 16, 11/54 patients (20.4%) had confirmed progression (Table 2). The 12-month progression rate was 37% (20/54 patients).

Seizures (including one absence) were identified as the second most frequent AE, occurring in 6/29 patients receiving antiepileptic treatment (cf. Table 1). All events were unrelated to the intervention and occurred outside of training sessions. No seizures were reported in patients without prior history of seizures, and all affected individuals remained seizure-free for the duration of the study following medication adjustments. Seizures did not result in exclusion from the study in any case.

Importantly, also no ERAEs led to premature study termination.

### Cardiorespiratory Fitness

**Figure 3.**
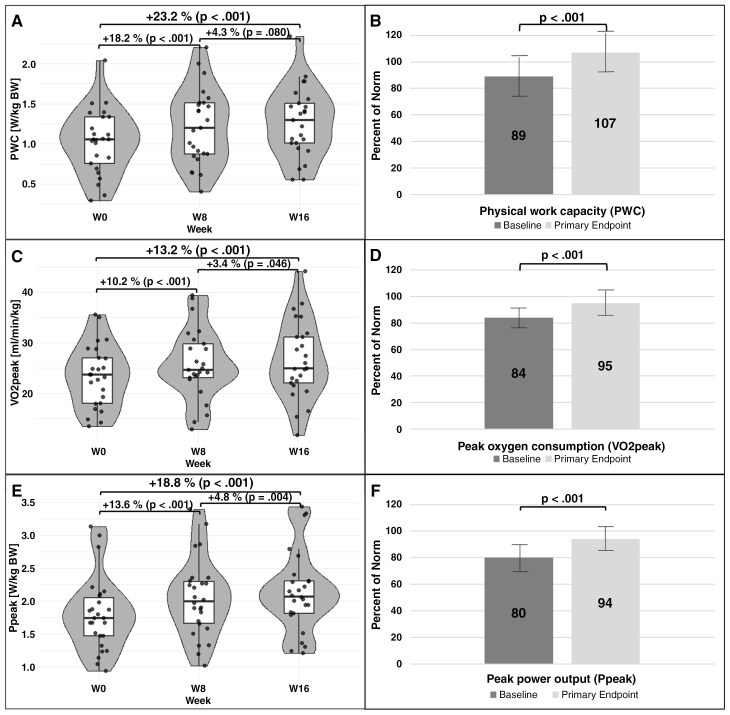
Primary and Key Secondary Performance Endpoints. PWC_75%_ (A and B), VO_2_peak (C and D), and Ppeak (E and F). Data shown are from all patients who completed week 16 only (*n* = 28). Baseline means were as follows: PWC_75%_ = 1.023 W/kg BW, VO_2_peak = 23.04 ml/min/kg, and Ppeak = 1.771 W/kg BW. As the data were normally distributed, paired *t*-tests were performed to assess significant changes over time. Significant improvements were observed between week 0 and week 8, likewise between week 0 and week 16, for all parameters (A, C, and E). Smaller, yet significant changes were observed between weeks 8 and 16 in most parameters. In addition, physical performance values are presented as percentages of the norm (B, D, F). At baseline, patients exhibited scores below their norm. At week 16 (primary endpoint) all parameters approached or surpassed the corresponding normative value (norm values are based on Finger et al.^23^ (B) and Gläser et al.^34^ (D and F). The bars represent the 95% confidence interval.

By week 16, all measured key fitness parameters (PWC_75%_, VO_2_peak, and Ppeak) had significantly improved (cf. Figure 3A, C, and E). The largest gains occurred between weeks 0 and 8, with smaller, yet mostly still significant, improvements from weeks 8 to 16. By week 16, patients had approached normative values (cf. Figure 3B, D, and F).

#### ECOG stratification.

—Both ECOG groups showed significant improvements in cardiorespiratory fitness from baseline to week 16 (Supplementary Figure 3A and Supplementary Table 2). ECOG 0 patients exceeded their adjusted norm for VO_2_peak, whereas ECOG 1 patients, although starting from much lower baseline values, were still able to increase their VO_2_peak. Further parameters are reported in Supplementary Table 2.

#### Activity level group stratification.

—As outlined in the methods, patients were divided into 3 different post-diagnosis activity groups according to their sporting behavior: all-inactive, get-inactive, and stay-active. All activity groups benefited from the exercise program, with significant improvements regardless of their initial post-diagnosis activity levels (Supplementary Figure 4A and Supplementary Table 3).

Further results on performance development, including analyses on mid-intervention (completers of week 8, *n* = 38) and follow-up (completers of week 24, *n* = 23), are provided in the Supplement. In short: Already at mid-intervention, performance changes across all parameters reached statistical significance (*p* < .001, all comparisons). These improvements were consistent when expressed as percentages of age- and sex-adjusted normative values (Supplementary Table 4 and Supplementary Figure 5). At follow-up, most fitness parameters were sustained or slightly declined, without significant changes compared to week 16 (Supplementary Figure 6).

### Quality of Life

**Figure 4.**
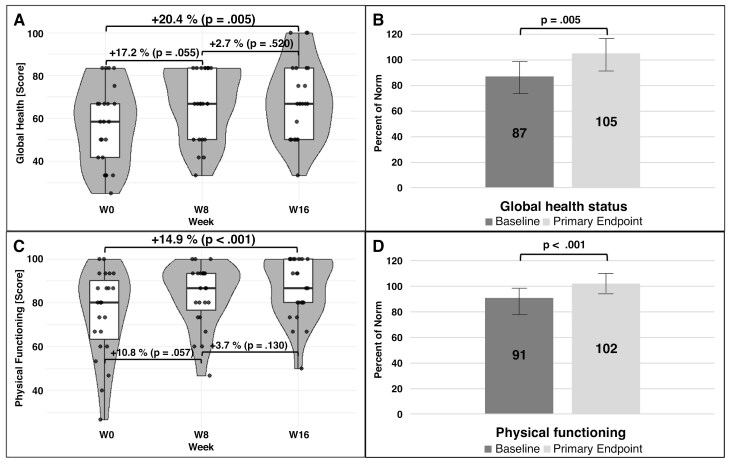
Changes in Global Health (A and B) and Physical Functioning (C and D) Throughout the Intervention. Data shown are from all patients who completed week 16 only (*n* = 28). Global health status (A) and Physical functioning scores (C) (baseline mean scores: 56.9 and 75.1) improved significantly between week 0 and week 8, with smaller, non-significant changes between weeks 8 and 16. In B and D, QoL values are represented as percentages of the norm. Norm values are based on a large European cohort (*n* = 11,343 participants from 11 EU countries) as reported by Nolte et al.^36^ In both panels B and D, the bars represent the 95% confidence interval. Data were tested for normal distribution using the Shapiro-Wilk test at each time point, with either a paired *t*-test or Wilcoxon test applied to assess significant changes over time.

QoL, assessed via global health status and physical functioning, improved significantly by week 8 and remained stable through week 16, reaching a “plateau level” (Figure 4A and C). Initially below normative values, both QoL domains normalized after 16 weeks of intervention (Figure 4B and D). By week 24 (follow-up), these effects persisted (Supplementary Table 5).

Correlation analyses showed that both the QoL measures and the physical fitness measures showed a strong correlation (Supplementary Figure 7). Physical functioning correlated well with both fitness parameters; correlations with global health status nearly reached statistical significance.

### Predictors of Response

Exploratory analyses investigating baseline characteristics as potential predictors of intervention response (age, sex, MGMT promoter methylation status, time of inclusion, travel burden, ECOG, dexamethasone use, antiepileptic drug use, presence of brain tumor-specific symptoms, neurological impairment) did not reveal any clinically meaningful associations with improvements in physical performance or QoL (Supplementary Table 7).

Furthermore, patients with disease progression by week 16 did neither show a distinct early decline nor a non-response to exercise in fitness or global health score compared to those without progression (*p* > .05, all comparisons; data not shown).

## Discussion

This study prospectively evaluated the feasibility, safety, and efficacy of an intensive 16-week supervised exercise program in surgically treated HGG patients undergoing adjuvant chemotherapy. The findings are clinically relevant, as they yield direct benefits to brain tumor patients by dispelling safety concerns, supporting feasibility, and demonstrating effectiveness of intensive training in improving physical fitness and QoL.

### Feasibility and Safety

Most importantly, the intervention was safe and caused no harm. Although AEs were reported in 72% of patients, the vast majority (80%) were unrelated to the exercise intervention, highlighting its safety. The rate of ERAEs was low (14 events (20%), 13/14 grade 1, 1 grade 2), all events resolved immediately and never led to study termination. Our ERAE numbers are in line with the BRACE study^10^ (9 ERAEs in 12 patients, grade 1 and 2 only), which is the only other exercise trial in brain tumor patients that recorded AEs that meticulously both during and outside of exercise sessions throughout the whole study conduct.

Disease progression was the leading cause of AEs. Given its high incidence in HGG within the first year, we aimed to rule out any signal of exercise-induced acceleration. Due to the lack of direct comparators, we decided to compare our 12-month progression rate with the well-known clinical trial by Herrlinger et al.^33^ due to the similarity in patient characteristics (eg, age, KPS ≥ 70, and IDH mutation rates). They reported a 12-month progression rate of approx. 40% in MGMT promoter-methylated patients. Within this molecularly defined subgroup, we observed progression in 8 out of 33 patients (26%). Across our entire study cohort, regardless of MGMT status, 20 out of 54 patients (37%) showed confirmed progression at 12 months. Therefore, we assume that intensive exercise does not accelerate disease progression.

Seizures, the second most common AE, exclusively occurred in some patients with a history of epilepsy and were always unrelated to training sessions. This demonstrates that intensive exercise does not provoke seizure activity in this cohort, consistent with findings from van den Bogard et al. on exercise in individuals with epilepsy.^37^

The feasibility results, however, demonstrate both strengths and challenges in implementing a structured exercise intervention for HGG patients. While the accrual rate of 28% met the predefined target, significant barriers to study participation remained evident. Logistical issues, mainly travel distances, accounted for the majority of nonparticipation cases, a challenge also reported in comparable investigations.^9,38,39^ Notably, the accrual rate could have exceeded 50% if logistical barriers were mitigated. Integrating decentralized or home-based training models could be a measure to counteract these barriers. Alternatively, initiating the training sessions already in conjunction with routine radiotherapy appointments in the adjuvant concomitant radiochemotherapy treatment phase may further enhance participation rates—a strategy that has already proven successful in previous studies.^11,40,41^

Adherence was high, with a median session completion rate of 85%. Most patients (84%) also achieved the targeted training intensity (≥70% HRmax), supporting the feasibility of such intensive exercise, even during ongoing chemotherapy.

While the overall attrition rate of 48% exceeded our predefined threshold, further analysis revealed important subgroup differences, as baseline dexamethasone use turned out to be the major predictor of attrition. Specifically, among patients without dexamethasone at training commencement, 22 of 30 (73.3%) completed the study through week 16, resulting in an attrition rate of 26.7%. In this subgroup, inability to adhere accounted for only 3 of 30 drop-outs (10%). In contrast, among patients on dexamethasone at training commencement, only 6 of 20 (30%) completed the intervention (attrition rate 70%), with inability to adhere being the most frequent cause for discontinuation. These findings are highly relevant, as they suggest that for patients with stable disease throughout the study and not receiving corticosteroids at intervention start, the intensive program was feasible in nearly all cases—regardless of other baseline characteristics. In contrast, dexamethasone use emerged as a key factor affecting feasibility. While this was not initially anticipated, this finding warrants further investigation, and highlights the value of tailoring exercise interventions based on individual treatment profiles. Taken together, despite various challenges, the intervention was deemed overall feasible — particularly for patients without corticosteroid use at baseline.

### Cardiorespiratory Fitness

Second, our physical fitness assessments using maximal CPET, the gold standard for assessing cardiorespiratory fitness, confirmed the trainability of HGG patients within the ActiNO program. The significant improvements in all measured parameters - particularly PWC_75%_, VO_2_peak, and Ppeak - demonstrated patients’ ability to adapt to structured, high-intensive training regimen. These findings confirm the observations and the resulting hypothesis from our previous retrospective analysis of exercise data in glioma patients,^7^ which proposed a targeted 15% improvement in PWC_75%_ performance. This hypothesis has now been successfully validated in a prospective setting and even exceeded, with an observed mean improvement of 23%. The response to the exercise intervention was independent of baseline functional status, pre-diagnosis activity level, or various other baseline characteristics.

### Quality of Life

Third, our study also demonstrated meaningful improvements in both global health status and physical functioning, strengthening the idea that exercise can positively impact QoL in heavily burdened glioma patients. Remarkably is the normalization of both domains by week 16, comparable to a large, healthy European cohort. This finding is crucial, as all therapeutic concepts aim to prolong survival while maximizing QoL in HGG patients, making QoL a key determinant of overall treatment. The observed strong relationships between VO_2_peak, Ppeak, and physical functioning highlight the role of improved cardiorespiratory fitness in driving functional QoL benefits. However, the interplay between fitness parameters and global health status requires a more holistic approach to fully address this complex domain, since supervised, intensive training comprises psychological and social support as well.

### Strengths and Limitations

This study has several notable strengths. It is the first investigation to demonstrate that HGG patients, mostly GBM, still undergoing chemotherapy, can safely engage in such an intensive exercise program, reaching maximal exertion. By including all patients with a KPS ≥ 70, selection bias was minimized and broader inclusion ensured. All surgically treated participants had completed concomitant radiochemotherapy contributing to a more homogeneous cohort.

The oligocentric design (4 centers) improves generalizability of our findings. Our primary endpoint, the improvement in physical performance, was assessed using the gold standard (maximal CPET) for measuring cardiorespiratory fitness. AEs were recorded meticulously and comprehensively, providing valuable and reliable safety data that are critical for future implementation in clinical practice. Remarkably, despite many challenges in conducting clinical trials during the COVID-19 pandemic, over 50 grade 4 glioma patients could be enrolled in this 1:1 in-person training program.

Notwithstanding these strengths, several limitations should be acknowledged. First, the single-arm design without a control group limits causal interpretation. Natural recovery processes—such as tapering of corticosteroids or resolution of radiation side effects—may have contributed independently to observed improvements. Further, while PWC_75%_ is a submaximal parameter and independent of learning effects, a minor degree of learning cannot be entirely excluded for maximal CPET-derived outcomes (VO_2_peak or Ppeak), although considered as the gold standard, particularly in the absence of a control group. Future randomized controlled designs are needed to more clearly isolate intervention effects. A further limitation is the relatively high drop-out rate, particularly during the first 8 weeks, which was largely attributed to the inability to adhere to the intensive exercise regimen among patients on dexamethasone. This raises questions about the broader feasibility of such an intensive exercise program for all HGG patients and suggests that an even more personalized and maybe less intense approach might be necessary to address a wider range of patients. Nonetheless, drop-out due to disease progression is likely unavoidable in this patient population, and should not preclude offering (intensive) exercise interventions, especially to those who are stable and motivated. Relatedly, the study sample reflects a positively selected subgroup with relatively high functional status, as required by the exercise protocol. While this was necessary to ensure participant safety and feasibility of the intensive training and CPET procedures, it limits generalizability. Future studies should aim to include more functionally diverse patient populations and explore more adaptable training formats.

Finally, while the 1:1 supervision format was critical to ensure safety and adherence in this vulnerable patient group, who had not previously been exposed to such intensive exercise in a clinical trial before, it also limits scalability and broader clinical implementation. This trial format requires considerable personnel resources, time, costs, and coordination, making it demanding for large-scale or routine application. Future research should therefore explore more accessible formats (eg, hybrid, group-based, or home-supported) and assess cost-effectiveness of structured exercise interventions. Given the encouraging clinical outcomes, expenses for supervised training as additional supportive care may be modest when compared to the costs of many oncologic treatments.

### Considerations for Future Studies

This study successfully demonstrated the trainability of glioma patients and the feasibility and safety of an intensive exercise regimen, providing a crucial foundation for future research. A key goal should now be to identify the most effective training parameters for improving QoL in brain tumor patients by establishing the FITT principles (frequency, intensity, time, and type).^17,42^ Our findings suggest that significant improvements occur primarily in the first 8 weeks, followed by a plateau, indicating that an optimal intervention duration may be shorter than 16 weeks.

Future studies should explore whether less intensive programs can yield comparable QoL benefits while reducing drop-out rates, thereby making interventions more accessible to a wider range of patients. Likewise, tailoring exercise regimens to individual capabilities is essential, as intensive training was feasible for the majority but too demanding for others, especially patients on dexamethasone. A controlled, randomized trial comparing high- and lower-intensity exercise regimens could clarify whether the fitness gains associated with high-intensity training are necessary for meaningful QoL improvements, particularly in managing fatigue and other treatment-related symptoms. Given its high burden among brain tumor patients, fatigue and further clinically relevant parameters (eg, cognitive function) should be considered as key outcome measures to better capture the broader benefits of exercise interventions in future studies. Determining the optimal intensity for this vulnerable population is a critical next step in refining exercise interventions for brain tumor patients.

## Conclusion

This study has demonstrated that supervised and structured high-intensity exercise enhances physical fitness and QoL of HGG patients undergoing adjuvant chemotherapy to a great deal and is safe. However, given the high influence of dexamethasone use on attrition rates, such intensive programs are not feasible for all patients with HGG. These findings underscore the need for more flexible, personalized exercise approaches that accommodate the heterogeneity of this population. To develop appropriate and quality-assured exercise therapies tailored to HGG-specific symptoms, a randomized controlled trial comparing different exercise regimens is the next apparent step to identify the optimal training criteria most beneficial to specific needs and symptom management.

## Supplementary material

Supplementary material is available online at *Neuro-Oncology* (https://academic.oup.com/neuro-oncology).

Full list of investigators (MMH study group):

(1) University of Münster:

Department of Neurosurgery, University Hospital: Dorothee Wiewrodt, Johanna Jost-Engl, Ralf Brandt, Maren Kloss, Nora Hansel

Pulmonary Research Division, Department of Medicine A, University Hospital: Rainer Wiewrodt, Johanna Jost-Engl, Leonora Grimm, Irmtraud Früchte

Institute of Sports Science: Klaus Völker, Ross Julian, Lothar Thorwesten

Institute of Biometry and Clinical Research, University Hospital: Joachim Gerß, Andreas Faldum

(2) Saarland University:

Department of Neurosurgery, University Hospital: Ralf Ketter, Steffi Urbschat, Joachim Oertel, Johanna Jost-Engl, Gabi Franke

Department of Pneumology, University Hospital: Philipp Lepper

(3) Ruhr-University Bochum:

Department of Neurosurgery, University Hospital: Kathleen Jetschke, Sylvia Rekowski

(4) University of Cologne:

Department of Neurosurgery, Faculty of Medicine and University Hospital: Carolin Weiss Lucas, Sophia Kochs

Studygroup Oncological Exercise Medicine, Center for Integrated Oncology: Freerk Baumann

noaf176_Supplementary_Materials_1

## Data Availability

We will make our research data available upon reasonable request. Researchers interested in accessing the data can contact the corresponding author. Access will be provided in accordance with ethical and legal guidelines, subject to necessary approvals.
