# Supplementary material for: Enhancing cardiorespiratory fitness and quality of life in high-grade glioma through an intensive exercise intervention during chemotherapy: Proof of concept
Source: Neuro Oncol. 2025 Jul 25;27(11):2977–90. doi: 10.1093/neuonc/noaf176 (PMC12908479; doi:10.1093/neuonc/noaf176)
Supplement: noaf176_Supplementary_Materials_1 [file noaf176_supplementary_materials_1.pdf]

SUPPLEMENT
TO:

Enhancing Cardiorespiratory Fitness and Quality of Life in High-Grade Glioma through an Intensive Exercise Intervention during Chemotherapy: Proof of Concept

Johanna Jost-Engl[1,2,3], Ralf Ketter[2], Ralf Brandt[1], Klaus Völker[4], Joachim Gerß[5], Kathleen Jetschke[6], Carolin Weiss Lucas[7], Freerk T. Baumann[8], Philipp M. Lepper[9,10], Steffi Urbschat[2], Walter Stummer[1], Rainer Wiewrodt*[3], and Dorothee Wiewrodt*[1], for the MMH Trial Investigators†

*equally contributed

† The full list of Investigators can be found in the Acknowledgement section.

Table of Contents:

Supplementary Content 1: Rationale for hypothesis of expected improvement in $PWC_{75\%}$

Supplementary Content 2: Eligibility Criteria

Tables

Supplementary Table 1: Adverse events by system organ class and CTCAE severity grades (percentages based on total number of AEs)

Supplementary Table 2: ECOG-stratification for changes in physical fitness and QoL parameters from baseline (W0) to primary endpoint (W16) in patients with complete datasets (n=27)

Supplementary Table 3: Changes in physical fitness and QoL parameters across different activity level groups from baseline (W0) to primary endpoint (W16)

Supplementary Table 4: Mid-intervention analysis of physical performance key metrics

Supplementary Table 5: Follow-up data of global health and physical functioning scores

Supplementary Table 6: Associations between dexamethasone use (at training commencement) and patients characteristics (A), and intervention-dependent variables (B)

Supplementary Table 7: Exploratory analysis of baseline characteristics as predictors of intervention response (high vs. low responders based on median split of delta scores)

Figures

Supplementary Figure 1

Supplementary Figure 2: Recruitment process at the University Hospital Münster

Supplementary Figure 3: Development of physical fitness and QoL, stratified by ECOG

Supplementary Figure 4: Development of physical fitness and QoL, stratified by activity level groups

Supplementary Figure 5: Changes in key performance metrics between baseline (W0) and mid-intervention (W8)

Supplementary Figure 6: Development of physical fitness parameters across the study period, including follow-up at week 24 (n=23)

Supplementary Figure 7: Correlations between QoL and physical fitness parameters

Supplementary Figure 8: Flow of participants according to dexamethasone use at study consent (n=54; n=24 on dexa, median dose 2mg/day) and at training commencement (n=50; n=20 on dexa, median dose: 3 mg/day) and subsequent changes over the study period

Supplementary Figure 9: Relationship between dexamethasone use at training commencement and reasons for study termination (n=54)

Supplementary Content 1: Rationale for hypothesis of expected improvement in $PWC_{75\%}$

The hypothesis of a ≥15% improvement in $PWC_{75\%}$ as the primary outcome of the ActiNO study was derived from two main sources:

(1) Prior retrospective data from our own group

In our previous retrospective analysis ((7), Jost et al., 2023), which involved 45 glioma patients (58% glioblastoma), an average increase of 11.5% in $PWC_{75\%}$ was observed following a median of 16 training sessions. These sessions were conducted in a less structured setting (i.e., no fixed schedules, heterogeneous inclusion points, and tailored based on clinical needs outside of a prospective study framework). Notably, patients with a higher number of training sessions in that cohort tended to demonstrate even larger improvements. Based on these observations, we expected that a prospective, highly standardized intervention design with a longer intervention period would enable further performance improvements, making a ≥15% increase a reasonable and clinically meaningful target.

(2) Evidence from randomized controlled exercise trials in oncology

A large meta-analysis by Scott et al. (28) covering 48 randomized controlled trials in various cancer populations (mostly breast cancer) showed that exercise interventions led to significant improvements in VO_2peak (+2.8 mL/kg/min vs. control, $p<0.001$). Referencing the normative VO_2peak values from the ACSM guidelines (29) for the relevant patient age group (mean ~60 years; normative range ~25.8–31.8 mL/kg/min), this improvement corresponds to an approximate relative increase of ~10%. Considering the good test-retest reliability of VO_2peak (correlation coefficient ~0.92) and its strong correlation ($r = 0.69–0.98$) with submaximal measures[1,2], we estimated that a 10% VO_2peak increase would correspond to a 15% $PWC_{75\%}$ increase. This estimate was further supported by internal data analyses from >100 prior datasets, indicating that relative improvements in $PWC_{75\%}$ tend to exceed VO_2peak gains, with an observed $\Delta PWC_{75\%} / \Delta VO_2$peak ratio of 1.56.

Taken together, these prior observations and external benchmarks provided the rationale for our hypothesis that a 15% improvement in $PWC_{75\%}$ over the 16-week intervention period would be both feasible and clinically relevant for our patient cohort.

[1] Noonan V, Dean E. Submaximal exercise testing: clinical application and interpretation. Phys Ther. 2000;80(8):782–807.

[2] Eng JJ, Dawson AS, Chu KS. Submaximal exercise in persons with stroke: test-retest reliability and concurrent validity with maximal oxygen consumption. Arch Phys Med Rehabil. 2004;85(1):113–8.

Supplementary Content 2: Eligibility Criteria

Inclusion Criteria:

- Newly diagnosed glioblastoma (according to 2016 WHO classification; inclusion until 12 August 2022)*
- Newly diagnosed high-grade glioma (WHO grade 3 or 4; inclusion from 12 August 2022, following ethics amendment)*
- Karnofsky Index ≥ 70
- Eastern Cooperative Oncology Group (ECOG) < 2
- ≥ 18 years
- Completed surgical therapy
- Completed radiation and chemotherapy
- Thrombocytes > 50.000/µl
- Hb > 8 mg/dl
- Ability to give consent
- Mother tongue German/very good German skills

Exclusion Criteria:

- Diagnosed dementia (Mini-Mental-State-Test < 24/30 points)
- Pain (strong, permanent, restricting movement)
- Impairment of consciousness (e.g., due to elevated intracranial pressure)
- Fever
- Acute infection
- Pregnancy and lactation
- Insufficiently adjusted epilepsy (despite anticonvulsive therapy > 3 focal seizures per day or > 1 generalized seizure in the previous 3 days)
- Inability to follow exercise instructions (e.g., due to neurocognitive impairment)
- Contraindications to intensive physical exertion or cardiopulmonary exercise testing (CPET)

Notes:

* Due to the new 2021 WHO classification, eligibility criteria were amended in consultation with the ethics committee on August 12, 2022. Initially, only patients with glioblastoma (based on the 2016 WHO classification) were included. Following the amendment, inclusion was broadened to patients with malignant gliomas (WHO grade 3 or 4) who had received radiochemotherapy.

Supplementary Table 1: Adverse events by system organ class and CTCAE severity grades (percentages based on total number of AEs)

System organ class	Any Adverse event n (%)*	Adverse event (AE)	Total AE n (%)*	Unrelated AEs			Treatment-related AEs (◊Possibly / □Definitely)		
				Grade 1 n (%)*	Grade 2 n (%)*	Grade 3 n (%)*	Grade 1 n (%)*	Grade 2 n (%)*	Grade 3 n (%)*
**Nervous System**	**24 (37.5)**	Seizure	6 (9.4)	2 (3.1)	-	4 (6.3)	-	-	-
		Dizziness [a]	5 (7.8)	-	-	-	5 (7.8)◊□	-	-
		One-sided muscle weakness	4 (6.3)	1 (1.6)	3 (4.7)	-	-	-	-
		Concentration impairment	3 (4.7)	1 (1.6)	2 (3.1)	-	-	-	-
		Cognitive disturbance	2 (3.1)	-	1 (1.6)	1 (1.6)	-	-	-
		Dysphasia	2 (3.1)	-	2 (3.1)	-	-	-	-
		Headache	1 (1.6)	1 (1.6)	-	-	-	-	-
		Stroke	1 (1.6)	-	-	1 (1.6)	-	-	-
**General disorders**	**17 (26.6)**	Disease progression [b]	11 (20.4)	n.a.			n.a.		
		Fatigue [c]	5 (7.8)	2 (3.1)	3 (4.7)	-	-	-	-
		Localized edema (knee)	1 (1.6)	-	-	-	1 (1.6)□		
**Infection**	**6 (9.4)**	Upper respiratory	4 (6.3)	4 (6.3)	-	-	-	-	-
		COVID-19	1 (1.6)	1 (1.6)	-	-	-	-	-
		Other (unknown)	1 (1.6)	-	1 (1.6)	-	-	-	-
**Injury**	**6 (9.4)**	Fall	4 (6.3)	1 (1.6)	1 (1.6)	2 (3.1)	-	-	-
		Fracture	2 (3.1)	-	1 (1.6)	1 (1.6)	-	-	-
**Musculoskeletal**	**5 (7.8)**	Buttock pain [d]	3 (4.7)	-	-	-	3 (4.7)□	-	-
		Muscle-weakness lower limb	1 (1.6)	-	1 (1.6)	-	-	-	-
		Muscle-weakness trunk	1 (1.6)	-	1 (1.6)	-	-	-	-
**Cardiac**	**4 (6.3)**	Sinus bradycardia	2 (3.1)	-	-	-	2 (3.1)□	-	-
		Chest pain	1 (1.6)	-	-	-	1 (1.6)□	-	-
		Other: ST-segment depression [e]	1 (1.6)	1 (1.6)	-	-	-	-	-
**Gastrointestinal**	**1 (1.6)**	Vomiting	1 (1.6)	-	-	-	1 (1.6)◊	-	-
**Respiratory**	**1 (1.6)**	Bronchial obstruction	1 (1.6)	-	-	-	-	1 (1.6)□	-
**TOTAL**	**64 (100)**		**64 (100)**	**14 (21.9)**	**16 (25.0)**	**9 (14.1)**	**13 (20.3)**	**1 (1.6)**	**0 (0.0)**

*Percentages are based on the total number of AEs (n=64), representing 100%.

[a] In 2/5 patients, strength training exercises, especially when performed in a standing position (here: cable machine), sometimes led to intermittent dizziness, which was effectively managed by incorporating seated rest periods between sets, allowing further execution to remain feasible and well-tolerated. 1/5 experienced overexertion during endurance training, 1/5 experienced dizziness following maximal CPET, and 1/5 dizziness case was associated with documented cardiac issues. However, all events resolved without an intervention needed.
[b] Disease progression within the study protocol. In 10/11 cases (91%), progression was accompanied by clinical deterioration of general condition. In these cases, general clinical deterioration or the exacerbation of previously existing symptoms related to disease progression were not further listed in this table.
[c] Fatigue was recorded only if newly onset or deterioration of a preexisting condition.
[d] Buttock pain was observed exclusively at one study site and linked to the according ergometer saddle. The ergometer was switched to prevent further discomfort.
[e] Asymptomatic & non-reproducible

Supplementary Table 2: ECOG-stratification for changes in physical fitness and QoL parameters from baseline (W0) to primary endpoint (W16) in patients with complete datasets (n=27). Values are presented as means (M) or medians (Mdn), with standard deviations (SD) or interquartile ranges (IQR), and percentage changes (%Δ) with corresponding p-values (based on t-tests or Wilcoxon tests, depending on data normality). Both ECOG groups, despite differences in functional status and baseline scores, showed clinically meaningful improvements in their physical fitness and QoL.

	ECOG 1 (n=7)					ECOG 0 (n=20)				
	Baseline (W0)		Prim. E. (W16)			Baseline (W0)		Prim. E. (W16)		
**MEASURE**	M	SD	M	SD	Δ (p)	M	SD	M	SD	Δ (p)
VO2peak [ml/min/kgBW]	17.9	6.1	19.8	7.0	+10.4% (=.050)	24.8	5.5	28.3	6.9	+14.0% (<.001)
VO2peak [% of norm*]	64.3	16.1	71.7	22.6	+10.6% (=.036)	91.2	14.4	102.5	16.7	+12.4% (<.001)
Ppeak [W/kg BW]	1.241 (Mdn)	0.35 (IQR)	1.362 (Mdn)	0.85 (IQR)	+9.8% (=.018)	1.921	0.55	2.271	0.59	+18.2% (<.001)
Ppeak [% of norm*]	58.6	11.0	71.5	16.3	+22.0% (=.012)	87.8	19.9	102.3	19.7	+16.5% (<.001)
Global Health [score]	52.8	13.6	66.7	14.9	+26.3% (.125)	58.3	20.0	69.1	19.7	+18.5% (.011)
Global health [% of norm**]	80.3	20.8	101.5	23.1	+26.4% (.125)	89.3	31.0	105.9	31.0	+18.6% (.011)
Physical functioning [score]	68.9	19.2	80.0	14.0	+16.1% (.046)	86.7 (Mdn)	26.7 (IQR)	93.3 (Mdn)	20.0 (IQR)	+7.6% (.004)
Physical functioning [% of norm**]	82.4	22.2	95.8	16.7	+16.3% (.045)	99.3 (Mdn)	33.1 (IQR)	107.5 (Mdn)	24.6 (IQR)	+8.3% (.003)

*The normative values for VO_2peak and Ppeak are based on the SHIP study (24).

**QoL parameter norms are based on Nolte et al. (26).

Supplementary Table 3: Changes in physical fitness and QoL parameters across different activity level groups from baseline (W0) to primary endpoint (W16).

Values are presented as means (M) or medians (Mdn), standard deviations (SD) or interquartile ranges (IQR), and percentage changes (%Δ) with corresponding p-values (based on t-tests or Wilcoxon tests, depending on normality of the respective data). All groups benefited from the exercise program, showing improvements in both physical fitness and QoL regardless of their initial post-diagnosis activity levels. As expected, participants who had previously engaged in physical activity but then stopped (get-inactive) showed the greatest fitness improvements upon resuming training. Those who remained some level of activity after diagnosis (stay-active group) had the highest baseline values but were still able to achieve further significant gains.

	All-inactive (n=10)					Get-inactive (n=9)					Stay-active (n=8)				
	Baseline (W0)		Prim. EP (W16)			Baseline (W0)		Prim. EP (W16)			Baseline (W0)		Prim. EP (W16)		
**MEASURE**	M	SD	M	SD	Δ (p)	M	SD	M	SD	Δ (p)	M	SD	M	SD	Δ (p)
VO2peak [ml/min/kgBW]	20.0	5.3	21.8	7.0	8.8% (.101)	21.7	5.1	25.0	4.8	14.9% (<.001)	28.3	5.9	32.8	7.6	15.6% (.009)
VO2peak [% of norm*]	74.8	20.8	81.7	26.9	9.2% (.075)	81.2	11.1	93.8	13.6	15.5% (<.001)	99.4	14.9	111.4	14.3	12.1% (.007)
Ppeak [W/kg BW]	1.496	0.34	1.786	0.46	19.3% (.005)	1.627	0.39	2.046	0.37	25.8% (<.001)	2.276	0.65	2.568	0.77	12.8% (.007)
Ppeak [% of norm*]	70.4	17.3	83.9	20.6	19.2% (.002)	74.7	16.8	94.0	17.3	25.8% (<.001)	98.7	23.2	107.8	27.1	9.2% (.015)
PWC$_{75\%HRmax}$ [W/kg BW]	0.841	0.28	1.034	0.25	22.9% (.005)	1.011	0.35	1.305	0.39	29.1% (.001)	1.243	0.48	1.450	0.56	16.6% (.012)
PWC$_{75\%HRmax}$ [% of norm*]	77.6	19.7	90.4	22.0	16.5% (.022)	84.1	31.4	105.4	31.6	25.3% (<.001)	109.6	33.3	128.6	41.9	17.3% (.012)
P at RER 1.0 [W/kg BW]	0.833 (Mdn)	0.17 (IQR)	0.885 (Mdn)	0.39 (IQR)	19.2% (.308)	1.010	0.27	1.261	0.31	24.9% (.012)	1.313	0.49	1.580	0.58	20.3% (.031)
P at RER 1.0 [% of norm]	n.a.	n.a.	n.a.	n.a.	n.a.	n.a.	n.a.	n.a.	n.a.	n.a.	n.a.	n.a.	n.a.	n.a.	n.a.
Global Health [score]	52.8	13.8	71.3	14.5	35.0% (.006)	58.3	18.4	62.5	17.3	7.2% (.321)	70.9 (Mdn)	52.1 (IQR)	66.7 (Mdn)	50.0 (IQR)	-5.9% (.131)
Global health [% of norm**]	81.1	21.7	109.4	22.7	34.9% (.006)	89.5	28.3	95.8	26.2	7.0% (.322)	92.5	40.0	109.6	40.5	18.5% (.055)
Physical functioning [score]	69.6	16.4	85.9	10.8	23.4% (.010)	83.4 (Mdn)	28.3 (IQR)	86.7 (Mdn)	25.0 (IQR)	4.0% (.071)	86.7 (Mdn)	38.3 (IQR)	96.7 (Mdn)	37.5 (IQR)	11.5% (.066)
Physical functioning [% of norm**]	81.2	19.5	100.1	12.6	23.3% (.011)	102.9 (Mdn)	33.0 (IQR)	104.8 (Mdn)	13.9 (IQR)	1.8% (.093)	91.4	33.0	101.2	26.5	10.7% (.033)

*The normative values for VO_2peak and Ppeak are based on the SHIP study (34), and $PWC_{75\%}$ norms are based on the DEGS1 study conducted by the Robert Koch Institute (23).
**QoL parameter norms are based on Nolte et al. (36).

Supplementary Table 4: Mid-intervention analysis of physical performance key metrics. Values are presented as means (M) or medians (Mdn), standard deviations (SD) or interquartile ranges (IQR), and percentage changes (%Δ) with corresponding p-values (p, based on t-test or Wilcoxon test).

	Mid-intervention completers (n=37)				
	Baseline (W0)		Mid-intervention (W8)		
**MEASURE**	M	SD	M	SD	Δ (p)
VO2peak [ml/min/kgBW]	23.3	5.8	25.4	6.7	9.0 % (<.001)
VO2peak [% of norm*]	87.3	17.7	95.0	21.8	8.8 % (<.001)
Ppeak [W/kg BW]	1.787	0.51	2.003	0.55	12.1 % (<.001)
Ppeak [% of norm*]	82.2	20.0	91.8	21.6	11.7 % (<.001)
$PWC_{75\%HRmax}$ [W/kg BW]	1.027	0.39	1.193	0.43	16.2 % (<.001)
$PWC_{75\%HRmax}$ [% of norm*]	90.7	29.4	104.2	35.5	14.9 % (<.001)
P at RER 1.0 [W/kg BW]	0.978 (Mdn)	0.43 (IQR)	1.078 (Mdn)	0.43 (IQR)	10.2 % (<.001)
P at RER 1.0 [% of norm]	n.a.	n.a.	n.a.	n.a.	n.a.

*The normative values for VO_2peak and Ppeak are based on the SHIP-study (34), and the $PWC_{75\%}$ norms are based on the DEGS1-study conducted by the Robert Koch Institute (23).

Supplementary Table 5: Follow-up data of global health and physical functioning scores.

Global Health Status				
**WEEK**	**W0**	**W8**	**W16**	**W24**
**M ± SD (n)†**	56.4 ± 17.7 (54)	66.1 ± 16.1 (38)	67.7 ± 17.7 (28)	68.0 ± 16.2 (23)
**M ± SD (n)***	58.8 ± 18.9 (23)	66.7 ± 17.3 (23)	68.4 ± 20.1[$] (23)	68.0 ± 16.2[$] (23)
**Physical Functioning**				
**WEEK**	**W0**	**W8**	**W16**	**W24**
**M ± SD (n) †**	75.2 ± 20.2 (54)	83.2 ± 15.5 (38)	86.3 ± 13.6 (28)	84.9 ± 17.0 (23)
**M ± SD (n)***	77.5 ± 19.3 (23)	83.5 ± 14.5 (23)	86.8 ± 15.1[$$] (23)	84.9 ± 17.0[$$] (23)

† refers to data from all patients who had measurements at each time point
* refers to data only from patients who completed all four time points
[$] not significant (p=0.603)
[$$] not significant (p=0.812)

Supplementary Table 6: Associations between dexamethasone use (at training commencement) and patients characteristics (A), and intervention-dependent variables (B)

Variable	Chi²	p-value
**(A) Baseline Patients Characteristics**		
Age group (median split)	1.227	0.268
Sex (female vs. male)	0.043	0.836
MGMT promoter methylation (non-methylated vs. methylated)	0.781	0.377
Time of inclusion (median split)	0.051	0.821
ECOG performance status (0 vs. 1)	2.675	0.102
Antiepileptic drug use (no vs. yes)	1.473	0.225
Brain tumor specific symptoms (none vs. present)	0.005	0.941
Neurological impairment (none vs. present)	0.781	0.377
Tumor laterality (left vs. right)	0.153	0.695
**(B) Intervention-Dependent Variables**		
Intervention completers (no vs. yes)	**8.984**	**0.003**
Any adverse event (intent-to-treat) (ever vs. never)	2.789	0.095
Exercise-related AE (intent-to-treat) (ever vs. never)	0.127	0.721
$\Delta PWC_{75\%}$, week 16 (low vs. high responder, median split)	1.321	0.250
ΔGlobal Health, week 16 (low vs. high responder, median split)	0.031	0.859

Supplementary Table 7: Exploratory analysis of baseline characteristics as predictors of intervention response (high vs. low responders based on median split of delta scores)

	**ΔPWC$_{75\%}$ Week 8 (n=38)**	**ΔPWC$_{75\%}$ Week 16 (n=28)**	**ΔGlobal Health Week 8 (n=38)**	**ΔGlobal Health Week 16 (n=28)**
**Baseline Characteristic**	Chi² (p-value)	Chi² (p-value)	Chi² (p-value)	Chi² (p-value)
Age group (median split)	0.925 (p=0.336)	0.540 (p=0.462)	0.267 (p=0.605)	3.235 (p=0.072)
Sex (male vs. female)	0.002 (p=0.968)	5.571 (p=0.018)	0.285 (p=0.594)	1.051 (p=0.305)
MGMT promoter methylation (no vs. yes)	0.015 (p=0.901)	0.910 (p=0.340)	0.259 (p=0.611)	0.340 (p=0.560)
Time of inclusion (median split)	0.002 (p=0.968)	0.133 (p=0.716)	3.895 (p=0.048)	0.087 (p=0.768)
Travel burden (median split)	0.185 (p=0.667)	0.181 (p=0.671)	0.784 (p=0.376)	0.034 (p=0.855)
ECOG performance (0 vs. 1)	0.607 (p=0.436)	0.516 (p=0.473)	2.362 (p=0.124)	0.140 (p=0.708)
Dexamethasone use at inclusion (no vs. yes)	1.145 (p=0.285)	1.321 (p=0.250)	0.512 (p=0.474)	0.031 (p=0.859)
Antiepileptic drug use (no vs. yes)	0.002 (p=0.968)	0.181 (p=0.671)	0.285 (p=0.594)	1.965 (p=0.161)
Brain tumor specific symptoms (none vs. present)	0.577 (p=0.447)	0.042 (p=0.838)	3.638 (p=0.056)	1.958 (p=0.162)
Neurological impairment (none vs. present)	0.537 (p=0.464)	0.619 (p=0.431)	0.784 (p=0.376)	1.308 (p=0.253)

Notes:

- Response was defined via a median split of delta scores (Δ) between baseline (week 0) and week 8 and week 16, respectively.
- High responders: Patients with Δ above median; Low responders: Δ below median.
- Chi²=Chi-square test for univariate associations (cross tabulations) between baseline characteristics and responder status was applied.
- Although a p-value <0.05 was observed for sex at week 16, with females more frequently classified as high responders in PWC$_{75\%}$, p=0.018), this finding at week 16 is based on low numbers (n=12

females at week 16) and was inconsistent across time points (week 8 vs. week 16). Furthermore, a linear regression model did not confirm this effect at week 16 (p=0.156). Taken together, these findings suggest that the apparent sex difference observed in the categorical responder analysis may not be robust and should be interpreted with caution. No consistent or clinically meaningful association between sex and training response could be confirmed across models in our study cohort.

- Multivariate regression exploratory analyses (binary model and linear model) confirmed that none of the examined baseline characteristics were associated with better response to intervention at both week 8 and week 16 (data not shown).

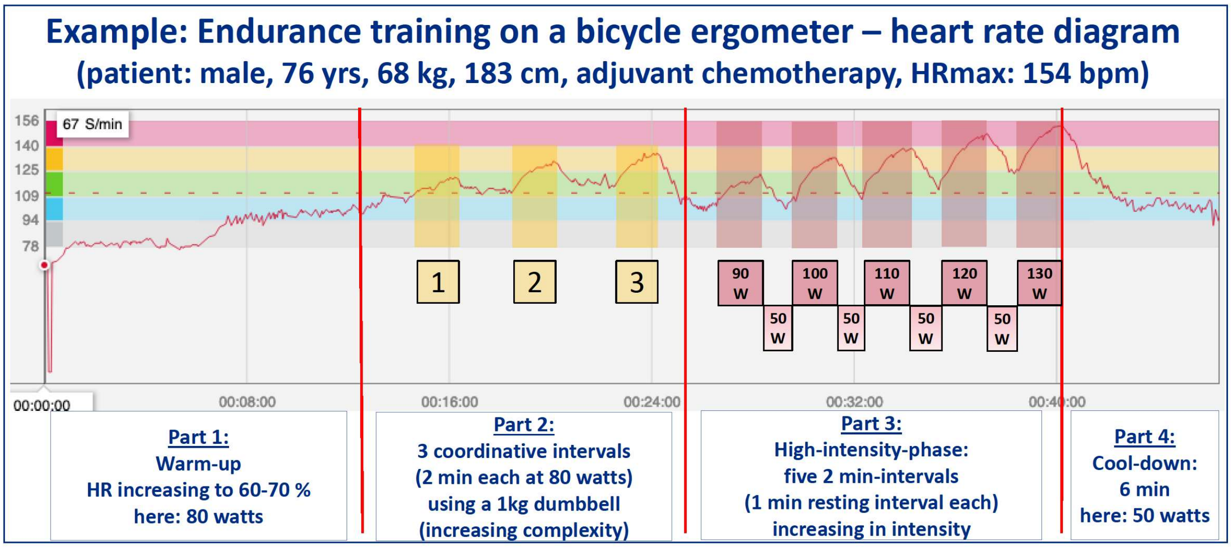

Supplementary Figure 1 displays a typical structure of an endurance training session, divided into four phases. The x-axis represents time in minutes, while the y-axis shows heart rate in beats per minute, continuously recorded using a Polar M430 and H7/9 sensor. Following the warm-up phase, additional coordinative elements are introduced alongside cycling (Phase 2). Submaximal exertion is achieved through a high-intensity interval segment (Phase 3, red boxes indicate actual workload), which is followed by a final cool-down phase (Phase 4). Throughout the entire session, an average heart rate of approximately 75% of the individual's HRmax is targeted. In summary, the exercise program in this study included structured, intensive endurance training, with an average intensity of ~75% HRmax (range 60% up to 90-95%) and high-intensity intervals exceeding 90% HRmax, positioning it within the intensive endurance training range.

This figure is reproduced from *Jost, J., Müther, M., Brandt, R. et al. Conceptual development of an intensive exercise program for glioma patients (ActiNO): summary of clinical experience. J Neurooncol **163**, 367–376 (2023). https://doi.org/10.1007/s11060-023-04354-y* under the terms of the Creative Commons Attribution 4.0 International License (http://creativecommons.org/licenses/by/4.0/).

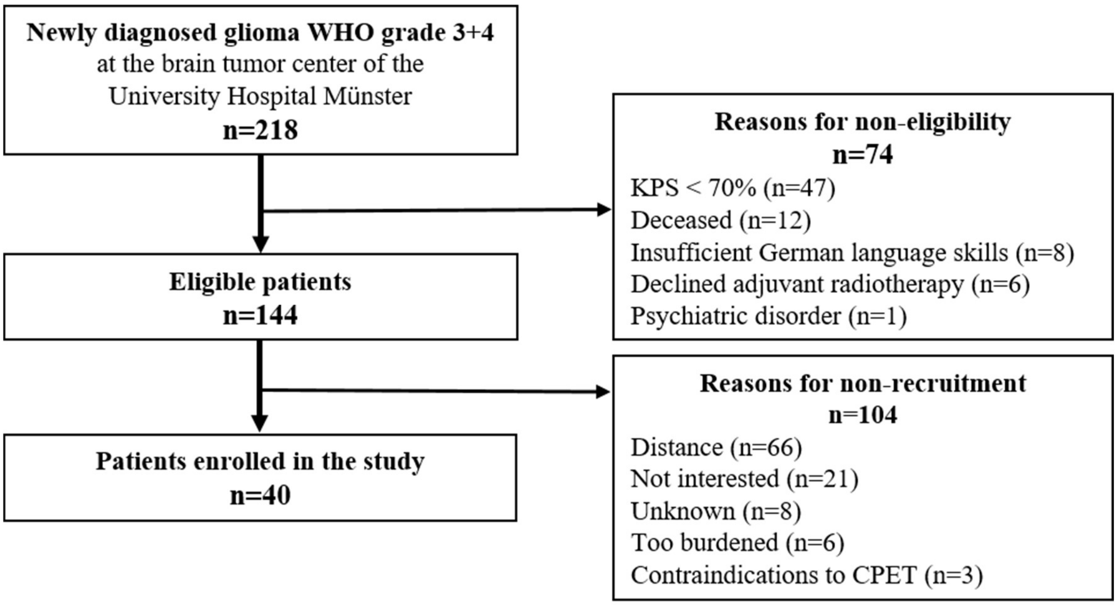

Supplementary Figure 2: Recruitment process at the University Hospital Münster.
A total of 218 patients with newly diagnosed WHO grade 3 or 4 glioma were screened for eligibility at the brain tumor center of the University Hospital Münster. Of these, 144 patients (66%) met the inclusion criteria and were deemed eligible for participation. Ultimately, 40 patients enrolled in the study (28% of eligible patients). The most common reason for ineligibility was a Karnofsky Performance Status (KPS) below 70. Among eligible patients, the by far most frequent reason for non-recruitment was the long distance to the training site, accounting for 63% of all non-recruited cases. Of note, as outlined in the main text, the total number of participants across all study sites was n=54 patients. As documentation of reasons for non-eligibility/non-recruitment for newly diagnosed WHO grade 3 or 4 glioma patients was outside of the study protocol, we could not expect detailed recruitment data from all sites. To reliably reflect actual screening processes, we decided to depict the accrual flow from the main study site only.

(This figure represents an updated version of the recruitment flowchart previously published in: Jost J, Völker K, Brandt R, Stummer W, Urbschat S, Ketter R, Wiewrodt D, Wiewrodt R; MMH Trial Investigators. Maximal cardiopulmonary exercise testing in glioblastoma patients undergoing chemotherapy: assessment of feasibility, safety, and physical fitness status. J Neurooncol. 2024 May;168(1):35-45. doi: 10.1007/s11060-024-04629-y)

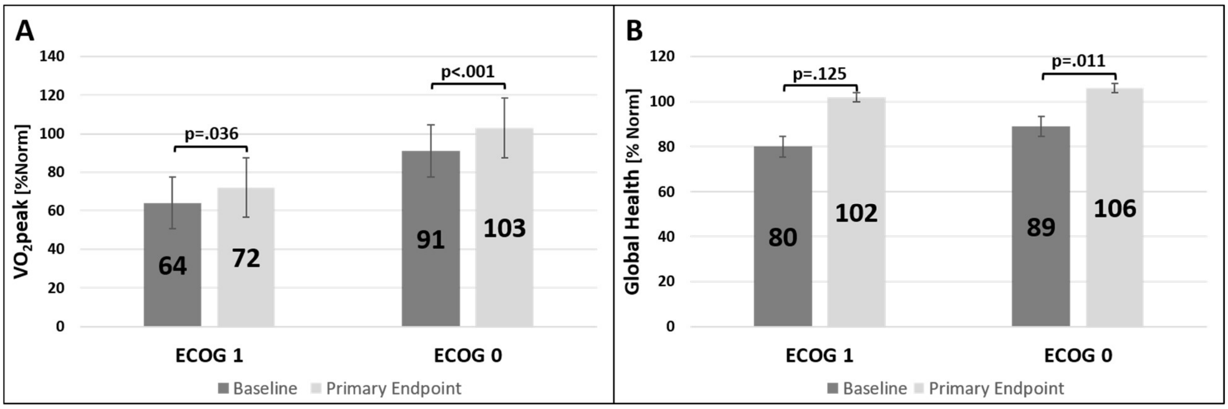

Supplementary Figure 3: Development of physical fitness and QoL, stratified by ECOG.
Data are presented as percentage of the norm with norm values based on Gläser et al. (34)(for VO_2peak, panel A) and Nolte et al. (36) (for Global Health, panel B). As both physical fitness data (A) and global health data (B) regarding ECOG were normally distributed, significant changes of mean values were calculated using t-tests. The bars represent the standard error.

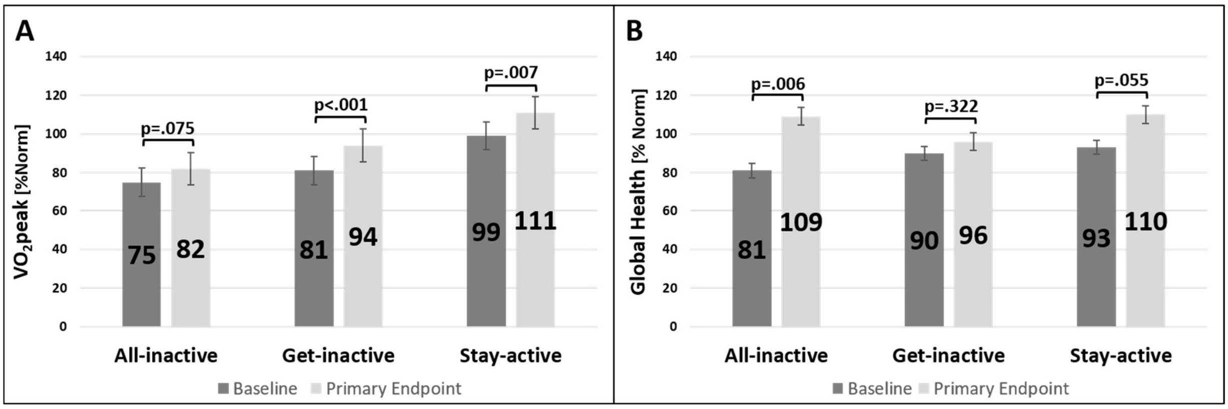

Supplementary Figure 4: Development of physical fitness and QoL, stratified by activity level groups.
Data are presented as percentage of the norm with norm values based on Gläser et al. (34)(for VO_2peak, panel A) and Nolte et al. (36) (for Global Health, panel B). All physical fitness data (A) and global health data (B) were normally distributed, except for stay-actives' global health (panel B, right columns). In the latter Wilcoxon test was applied, while other changes of mean values were calculated using t-tests. The bars represent the standard error.

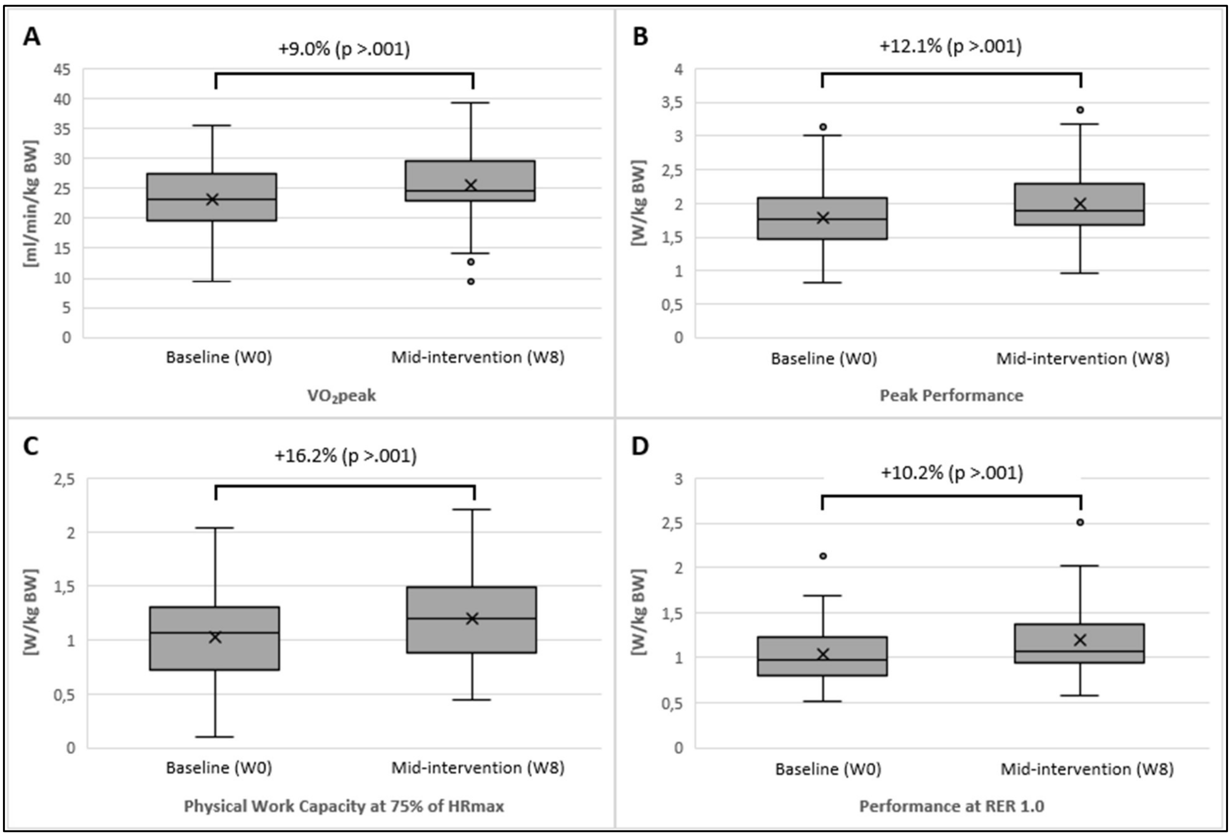

Supplementary Figure 5: Changes in key performance metrics between baseline (W0) and mid-intervention (W8) including all mid-intervention completers (n=37)**.** All values are presented as means or medians (depending on data normality) with corresponding percentage changes. T-tests were conducted for panels A-C, while Wilcoxon test was used for panel D.

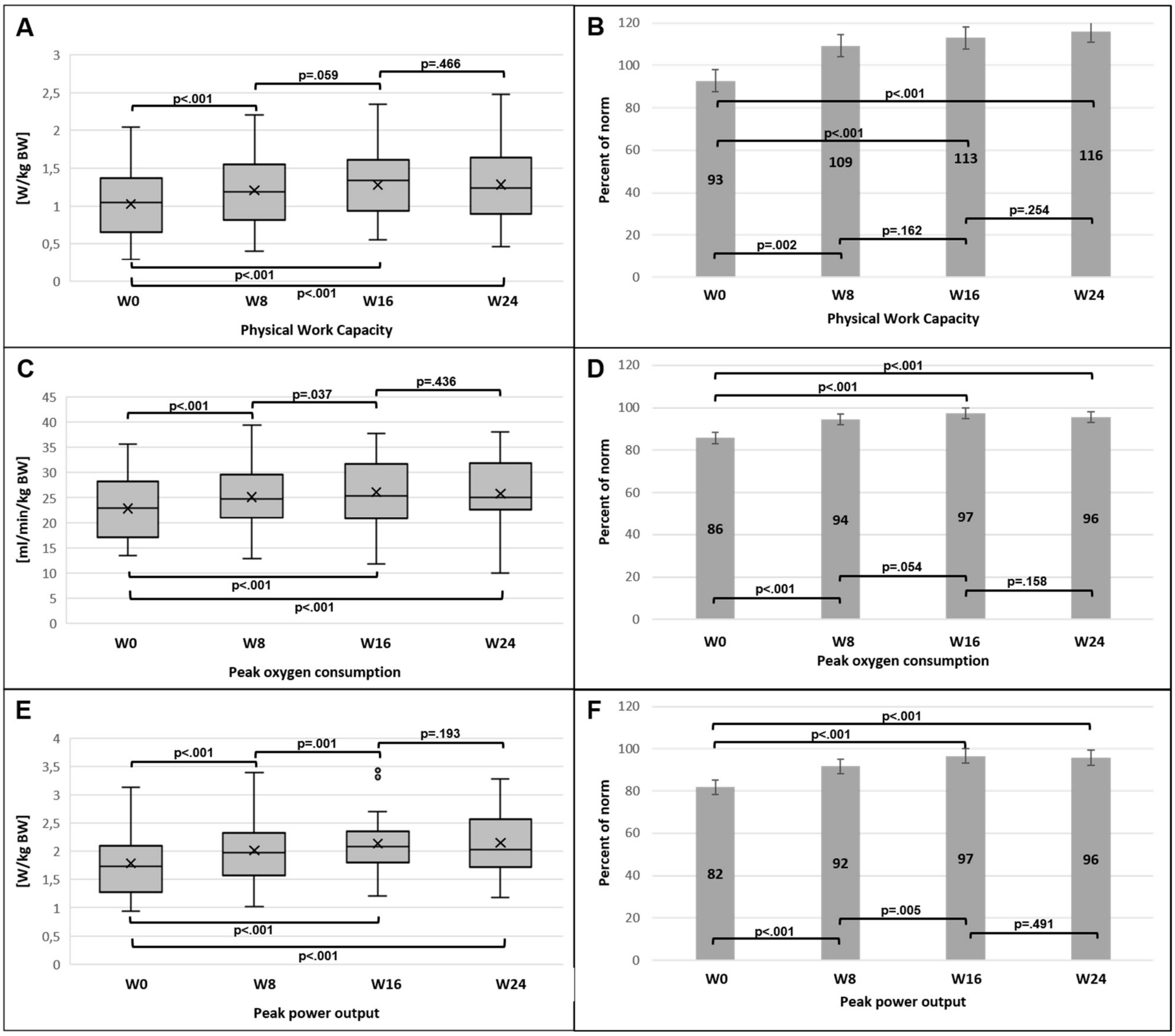

Supplementary Figure 6: **Development of physical fitness parameters across the study period, including follow-up at week 24 (n=23)**. Panels A, C, and E display absolute values for $PWC_{75\%}$ (A), VO_2peak(C), and Ppeak (E), while panels B, D, and F show these values as percentages of normative data. Normative data in both panels D and F are based on the SHIP-study (24), and in panel B on the DEGS1-study (25). Statistical significance was tested using paired t-tests for all comparisons, except in Panel D, where the Wilcoxon test was applied. Across most parameters, significant improvements were observed between baseline (W0) and mid-intervention (W8), as well as between W0 and W16. The follow-up measurements at W24 show sustained or slightly declining values for most fitness parameters, with no statistically significant differences compared to W16, suggesting a maintained effect of the intervention over time.

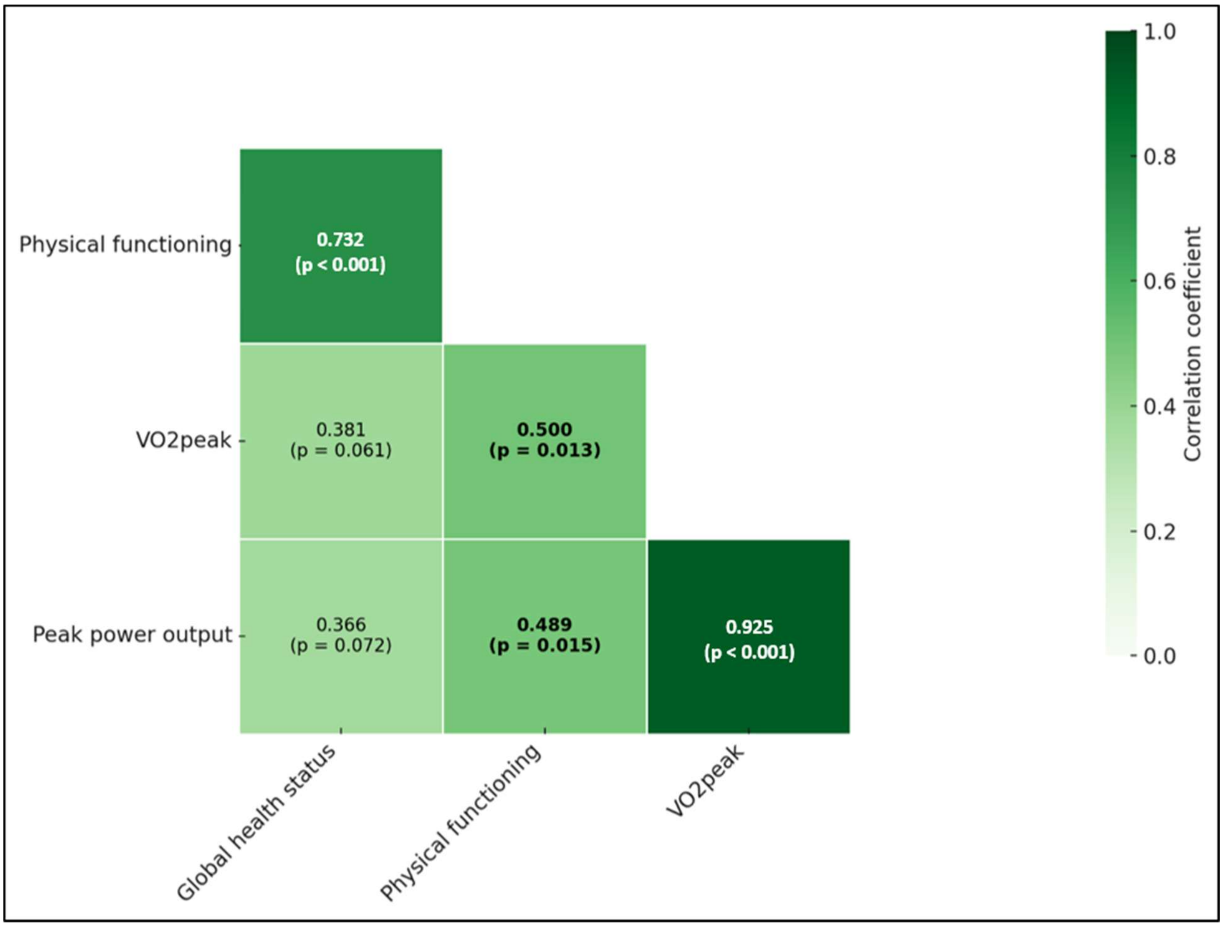

Supplementary Figure 7: **Correlations between QoL and physical fitness parameters** after 16 weeks of intervention (primary endpoint). The correlation matrix illustrates the relationships between QoL parameters (global health status and physical functioning) and physical fitness parameters (VO_2peak and Ppeak). Darker shades represent stronger correlations, while lighter shades indicate weaker correlations. Calculations were performed with Spearman-rank correlation tests.

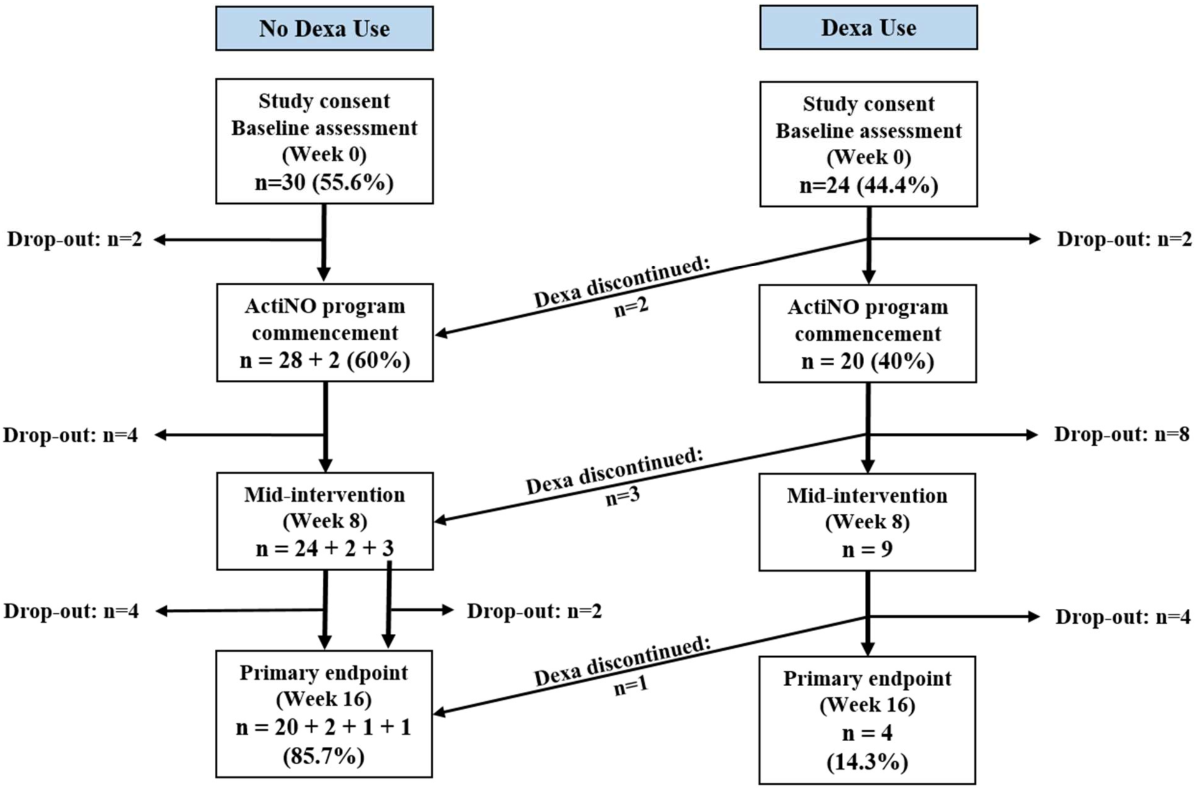

Supplementary Figure 8: Flow of participants according to dexamethasone use at study consent (n=54; n=24 on dexa, median dose 2mg/day) and at training commencement (n=50; n=20 on dexa, median dose: 3 mg/day) and subsequent changes over the study period.

<u>ActiNO-commencement until week 8:</u>

- In the no dexa group, 4 patients dropped out (13.3% of 30)
- In the dexa group, 8 patients dropped out (40.0% of 20)
- 3 patients in the dexa group discontinued dexamethasone
- Median dose among dexa patients at week 8: 2 mg/day

<u>By week 16:</u>

- In the no dexa group, 4 + 2 (formerly on dexa) additional patients dropped out
- In the dexa group, 4 additional drop-outs occurred
- 1 patient in the dexa group discontinued dexamethasone
- Median dose among dexa patients at week 16: 3 mg/day

<u>This results in a total of n=28 patients completing the study (baseline till week 16):</u>

- 20 patients (71.4%) who never received dexamethasone during the trial
- 4 patients (14.3%) who had received dexamethasone at baseline but discontinued it during the study (classified as dexamethasone-free at endpoint)
- 4 patients (14.3%) who were on dexamethasone throughout the whole study time at week 16
- Thus, 85.7% (24/28) of study completers were not receiving dexamethasone at the primary endpoint, compared to 55.6% (30/54) at baseline

Interpretation and Clinical Implications:

- This flow analysis clearly shows that baseline dexamethasone use was associated with a substantially higher drop-out rate.
- Baseline dexamethasone use may be a marker of reduced feasibility in exercise trials with high intensities and should be carefully considered when designing future interventions.
- Patients on corticosteroids may benefit from more flexible training options to support adherence.
- However, among those who remained in the study, successful participation was possible—even under corticosteroid therapy—highlighting that safety and benefit are still achievable.
- These findings underscore the importance of personalized approaches for exercise in neuro-oncology.

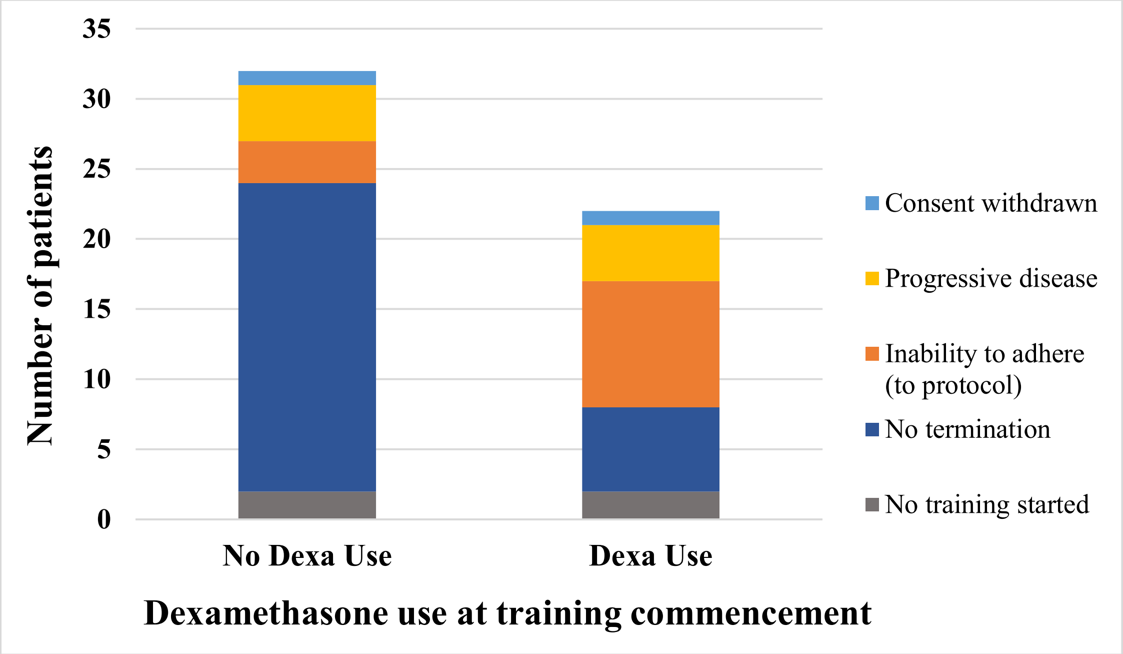

Supplementary Figure 9: Relationship between dexamethasone use at training commencement and reasons for study termination (n=54): This figure illustrates study completion status and reasons for early termination in patients stratified by dexamethasone use at baseline assessment. A clinically meaningful different pattern of study termination reasons was observed between groups (Pearson $Chi^2=10.656$, p=.031). Among participants not on dexamethasone (n=30+2 training not started), the majority completed the study (68.8%), with only 9.4% dropping out due to inability to adhere to the protocol, resulting in an attrition rate of 31.2%. In contrast, patients on dexamethasone (n=20+2 training not started) had a markedly higher attrition rate (72.7%), with 40.9% discontinuing due to inability to adhere. These findings suggest that dexamethasone use at baseline may negatively impact feasibility and should be explicitly considered in future trial designs.
